# Accuracy and efficiency of germline variant calling pipelines for human genome data

**DOI:** 10.1038/s41598-020-77218-4

**Published:** 2020-11-19

**Authors:** Sen Zhao, Oleg Agafonov, Abdulrahman Azab, Tomasz Stokowy, Eivind Hovig

**Affiliations:** 1 Department of Tumor Biology, Institute of Cancer Research, The Norwegian Radium Hospital, Oslo University Hospital, 0310 Oslo, Norway; 2 DNV GL, 1363 Høvik, Norway; 3 Center for Bioinformatics, Department of Informatics, University of Oslo, 0316 Oslo, Norway; 4 Division of Research Computing, University Center for Information Technology (USIT), University of Oslo, 0316 Oslo, Norway; 5 Computational Biology Unit, Institute of Informatics, University of Bergen, 5008 Bergen, Norway; 6 Department of Clinical Science, University of Bergen, 5021 Bergen, Norway

**Keywords:** Standardization, Clinical genetics, Genome, Genomics, Medical genetics, Sequencing, Genetics, Systems biology, Biological techniques, Bioinformatics, Genomic analysis, Sequencing, Computational biology and bioinformatics, Data processing, Standards

## Abstract

Advances in next-generation sequencing technology have enabled whole genome sequencing (WGS) to be widely used for identification of causal variants in a spectrum of genetic-related disorders, and provided new insight into how genetic polymorphisms affect disease phenotypes. The development of different bioinformatics pipelines has continuously improved the variant analysis of WGS data. However, there is a necessity for a systematic performance comparison of these pipelines to provide guidance on the application of WGS-based scientific and clinical genomics. In this study, we evaluated the performance of three variant calling pipelines (GATK, DRAGEN and DeepVariant) using the Genome in a Bottle Consortium, “synthetic-diploid” and simulated WGS datasets. DRAGEN and DeepVariant show better accuracy in SNP and indel calling, with no significant differences in their F1-score. DRAGEN platform offers accuracy, flexibility and a highly-efficient execution speed, and therefore superior performance in the analysis of WGS data on a large scale. The combination of DRAGEN and DeepVariant also suggests a good balance of accuracy and efficiency as an alternative solution for germline variant detection in further applications. Our results facilitate the standardization of benchmarking analysis of bioinformatics pipelines for reliable variant detection, which is critical in genetics-based medical research and clinical applications.

## Introduction

The innovation of next-generation sequencing (NGS) technologies has enabled exponential growth of the production of high throughput omics data^1–3^. Whole genome sequencing (WGS) and targeted whole exome sequencing (WES) are two main types of DNA sequencing protocols that have been broadly applied for the discovery of disease-related genes and identification of driver mutations for specific disorders^4–6^. In contrast to WES, WGS can assess all the nucleotides of an individual genome and allow detection of variants in both coding and non-coding regions. As a result of decreasing genome sequencing cost, WGS is becoming a powerful tool to investigate a wide range of complex inherited genetic diseases (e.g. heart disease, diabetes and psychiatric conditions), through the identification of causal germline variants^7–11^. The clinical application of WGS is gaining utility and consequently importance in underpinning personalized precision medicine^12,13^.

There is a necessity of bioinformatic pipelines for variant calling analysis on WGS data in a precise and efficient way prior to their integration into clinical diagnostic applications^14,15^. In general, a pipeline is comprised of the following steps: quality control, read alignment, variant calling, annotation, data visualization and reporting^12,16^. At the current stage of technological development, most of the clinical laboratories performing diagnostics of genetic disorders by WGS focus on two types of variants; single nucleotide polymorphisms (SNPs) and short insertions and deletions (indels). Many tools (e.g. Strelka, SpeedSeq, Samtools and Varscan2) have been developed for SNP and indel calling in the WGS analysis pipelines^17–20^. Among them, the Genome Analysis Toolkit (GATK) is one of the most used variant calling tools, as it applies a variety of state-of-the-art statistical methods (e.g. logistic regression, hidden markov model and naïve bayes classification) to accurately identify differences between the reads and the reference genome that are caused either by real genetic variants or by errors^21^. GATK can achieve high accuracy, but is still imperfect in memory management and running efficiency. Illumina has released a Dynamic Read Analysis for GENomics (DRAGEN) Bio-IT platform that provides an accurate and ultra-rapid solution for WGS data analysis^22^. The DRAGEN platform implements a highly configurable field-programmable gate array (FPGA) hardware technique to dramatically speed up analysis processes (e.g. alignment mapping and variant calling) and claims to do so without compromising accuracy. Verily Life Sciences (formerly Google Life Sciences) has developed DeepVariant for small germline variant detection based on a deep learning algorithm^23^. DeepVariant applies the python TensorFlow library to call variants in aligned reads by learning statistical relationships between images of read pileups around putative variants and true genotype calls. In 2016, the PrecisionFDA Truth challenge reported DeepVariant as the most accurate pipeline in the performance of SNPs calling^24^.

To compare the accuracy and efficiency of different variant calling pipelines and score their competence, it is critical to have high-quality benchmark datasets in which the true variant calls are well known. The Genome in a Bottle Consortium (GiaB) developed a golden callset (sample NA12878) that is widely used during development of variant calling pipelines and benchmarking^25^. Since its release, the NA12878 callset has been continuously upgraded as a comprehensive resource, and one of the major improvements was integration of the truth callset independently generated by the Platinum Genome (PG)^26^. An additional truth callset recently developed from a “synthetic-diploid” mixture of two haploid hydatidiform mole cell lines, CHM1 and CHM13, is now available in a public repository^27^. Although the variants in these two truth callsets represent real scenarios, the number of true variants is usually unknown, complicating its use for the assessment of accuracy (i.e. how close the defined truth callset is to the “true” mutational landscape). In contrast, simulated in silico WGS data allow users to generate variants under controlled scenarios with predefined parameters for which the “true” values are known, complementing the validation with real data. In several previous publications, performance comparisons of different variant calling pipelines (e.g. GATK, Samtools, Freebayes, SNVer and Stralka2), using both real and simulated WGS data, have been investigated, with results shown to vary according to the chosen pipelines and datasets to which they have been applied^23,24,27–34^. Until now, none of the studies have evaluated the three pipelines (GATK, DRAGEN and DeepVariant) together using multiple sets of WGS data for benchmarking. Importantly, by combining different datasets, the accuracy of genomic variant identification can be compared in a more systematic way, potentially providing a deeper understanding about their performance.

In this study, we obtained raw WGS data of NA12878 and “synthetic-diploid” samples from public repositories and constructed two sets of synthetic WGS data using a read simulator. A comprehensive benchmarking of GATK, DRAGEN, DeepVariant and their combinations was conducted using both real and simulated data. We aimed to evaluate the accuracy and efficiency of these pipelines for SNP and short indel detection, and identify the most precise and efficient combination of tools for small variant calling. These were assessed according to performance, concordance and time consumption, in order to provide a useful guideline of reliable variant identification for genetic medical research and clinical application.

## Materials and methods

### Sources of WGS benchmarking dataset acquisition

#### NA12878 (HG001) WGS data

The NIST reference material NA12878 (HG001) was sequenced at NIST, Gaithersburg, MD for the PrecisionFDA Truth Challenge. WGS library preparation was conducted using Illumina TruSeq (LT) DNA PCR-free sample Prep kits (FC-121-3001), and paired-end reads, insert size: ~ 550 bp were generated on HiSeq 2500 platform with rapid run mode (2 flow cells per genome). Raw paired-end fastq files (HG001-NA12878-50x_1.fastq.gz and HG001-NA12878-50x_2.fastq.gz) were obtained from https://precision.fda.gov/challenges/truth. In addition, another set of NA12878 raw WGS data sequenced in Supernat et al*.* was downloaded from the NCBI SRA repository (accession number: SRR6794144)^24^, using the SRA Toolkit.

#### “Synthetic-diploid” WGS data

Paired-end raw fastq files of “synthetic-diploid” WGS data were obtained from the European Nucleotide Archive (accession number: SAMEA3911976). The reference material, from a mixture of CHM1 (SAMN02743421) and CHM13 (SAMN03255769) cell lines at 1:1 ratio, was sequenced on HiSeq X10 platform using a PCR-free library protocol (Kapa Biosystems reagents)^27^. Two independently replicated runs, ERR1341793 (raw reads ERR1341793_1.fastq.gz and ERR1341793_2.fastq.gz downloaded from https://www.ebi.ac.uk/ena/browser/view/ERR1341793) and ERR1341796 (raw reads ERR1341796_1.fastq.gz and ERR1341796_2.fastq.gz downloaded from https://www.ebi.ac.uk/ena/browser/view/ERR1341796) were used for the benchmarking exercises.

#### Simulated WGS data

In addition to real WGS data, reads were synthesized in silico using the tool Neat-GenReads v2.0^35^. Briefly, two independent sets of simulated paired-end reads in fastq format, together with true positive variant datasets in VCF format, were generated from a random mutation profile (average mutation rate: 0.002) and a user defined mutation profile (using the golden truth callset assembled from CHM1 and CHM13 haploid cell lines), respectively. The simulation was performed on the basis of the human reference genome build GRCH37 decoy, with a read length of 150 bp, an average coverage of 40X, and a median insert size of 350 ± 70 bp.

### Implementation of variant calling pipelines

**Figure 1:**
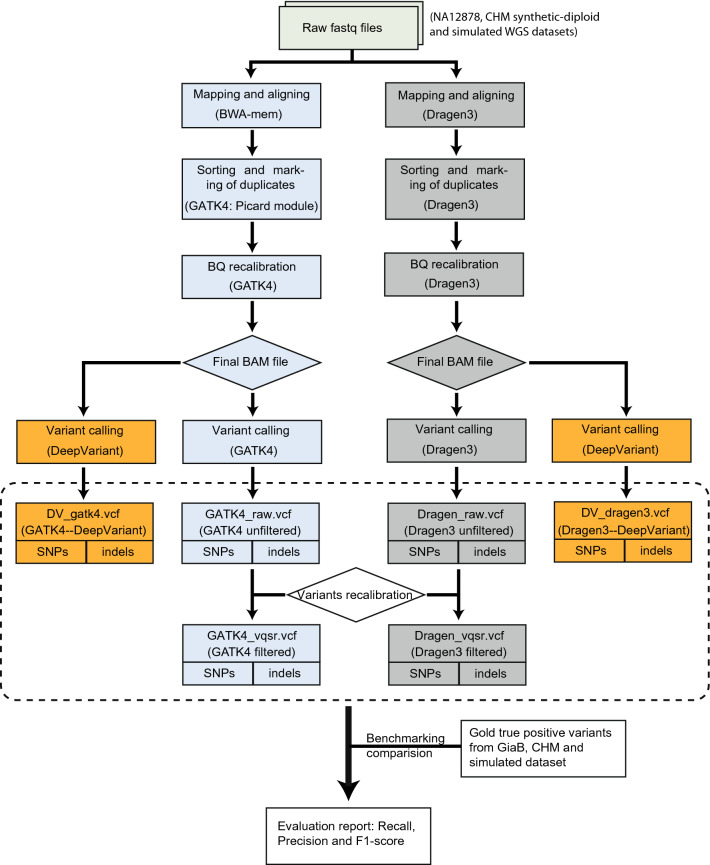
The flowchart of benchmarking analysis of different variant calling pipeline (GATK, DRAGEN and DeepVariant) combinations.

Germline variant calling was performed using the pipelines: (1) GATK v4.1.0.0^36^, (2) DRAGEN v3.3.11 and (3) DeepVariant v0.7.2 (see flowchart in Fig. 1)^23^.

The GATK pipeline workflow was applied following best practices (https://software.broadinstitute.org/gatk/best-practices). The raw paired-end reads were mapped to the GRCH37.37d5 reference genome by BWA-mem v0.7.15^37^. Aligned reads were converted to BAM files and sorted based on genome position after marking duplicates using Picard modules. The raw BAM files were refined by Base Quality Score Recalibration (BQSR) using default parameters. The variant calling (SNPs and indels) was performed with the HaplotypeCaller module. To speed up efficiency, the whole genome was split into 14 fractions and run in parallel, followed by merging of all runs into a final VCF file. Additionally, we used Variant Quality Score Recalibration (VQSR) to filter the original VCF files following GATK recommendations for parameter settings: HapMap 3.3, Omni 2.5, dbSNP 138, 1000 Genome phase I for SNPs training sets, and Mills- and 1000 Genome phase I data for indels.

The DRAGEN pipeline (https://www.illumina.com/products/by-type/informatics-products/dragen-bio-it-platform.html) followed a similar procedure as described for GATK best practices, including mapping and alignment, sorting, duplicate marking, haplotype calling and VQSR filtering.

The DeepVariant pipeline was run via a Singularity framework in accordance with online instructions (https://github.com/google/deepvariant). In general, this consisted of three steps: (1) make_example module—consumes reads and the reference genome to create the TensorFlow example for evaluation with deep learning models. (2) call_variants module—consumes TFRecord files created by the make_example module and evaluates the model on each example in the input TFRecord. (3) postprocess_variants module—reads the output TFRecord files from the call_variants module, combines multi-allelic records and writes out a VCF file. DeepVariant only used transformed aligned sequencing reads for variant calling, and so processed BAM file from GATK or DRAGEN pipelines was fed as input.

Six VCF files were finally generated per each WGS dataset; these represent different parameter settings and processing combinations of the pipelines in terms of their workflows, as depicted in Fig. 1 (i.e. *DV_gatk4*—GATK for BAM file and DeepVariant for variant calling; *DV_dragen3*—DRAGEN for BAM file and DeepVariant for variant calling; *GATK4_raw*—GATK for both BAM file and variant calling; *GATK4_vqsr*—callset from *GATK4_raw* filtered with VQSR; *Dragen3_raw*—DRAGEN for both BAM file and variant calling and *Dragen3_vqsr*—callset from *Dragen3_raw* filtered with VQSR). In addition, a merged VCF file was generated by combining the variants called by *DV_gatk4, DV_dragen3, GATK4_raw* and *Dragen3_raw* using bcftools v1.10.2^38^, and only variants called with the support of at least two pipelines were kept.

### Computing environment and resources

Variant calling processes were run both on a high-performance computing (HPC) cluster and on a local virtual machine (VM) within the sensitive data platform (TSD) at the University of Oslo. The settings of each node in the HPC cluster include 64 AMD CPU cores with a total size of 512 GB physical memory, a CentOS 7 operating system and a BeeGFS network file system. The FPGA hardware infrastructure was installed on one node specific for the DRAGEN pipeline application. The local VM had 40 CPU cores with a total 1.5 TiB physical memory, 2 TiB local disk with ext4 file system format and CentOS 7.

### Benchmark consensus of VCF files

The gold standard truth callset and high confidence genomic intervals (NIST v3.3.2) for the NA12878 (HG001) dataset were obtained from https://ftp-trace.ncbi.nlm.nih.gov/giab/ftp/release/NA12878_HG001/NISTv3.3.2/GRCh37/HG001_GRCh37_GIAB_highconf_CG-IllFB-IllGATKHC-Ion-10X-SOLID_CHROM1-X_v.3.3.2_highconf_PGandRTGphasetransfer.vcf.gz and https://ftp-trace.ncbi.nlm.nih.gov/giab/ftp/release/NA12878_HG001/NISTv3.3.2/GRCh37/HG001_GRCh37_GIAB_highconf_CG-IllFB-IllGATKHC-Ion-10X-SOLID_CHROM1-X_v.3.3.2_highconf_nosomaticdel.bed. To calculate the performance metrics, we used hap.py (version 0.3.8, vcfeval comparison engine) for comparison of diploid genotypes at the haplotype level https://github.com/Illumina/hap.py. The variant calling of WGS data from the mixture of CHM1 and CHM13 was compared to the “synthetic-diploid” benchmark truth callset and high-confidence regions (i.e. full.37d5.vcf.gz and full.37d5.bed.gz, which are included in the CHM-eval kit tool and available at https://github.com/lh3/CHM-eval, version 20180222) using vcfeval comparison engine^27^. For benchmarking variants identified in simulated WGS data, we performed a consensus evaluation against their truth positive callsets, both with and without high-confidence regions (i.e. HG001_GRCh37_GIAB_highconf_CG-IllFB-IllGATKHC-Ion-10X-SOLID_CHROM1-X_v.3.3.2_highconf_nosomaticdel.bed), respectively. The definitions of true positive (TP), false positive (FP) and false negative (FN) were based on the types of variant matching stringencies ”genotype match” (most strict—truth and query are considered as true positives when their unphased genotypes and alleles can be phased to produce a matching pair of haplotype sequences for a diploid genome) and ”local match” (less strict—truth and query variants are counted as true positives if their reference span intervals are closer than a pre-defined local matching distance)^39^. Precision, Recall and F1-score were calculated as TP/(TP + FP), TP/(TP + FN) and 2*TP/(2*TP + FN + FP), respectively.

### Definition of genome features for stratification analysis

Different types of genome contexts and biological features were applied in stratification analysis^33^. (1) Low complexity regions: ‘*_merged_slop5.bed.gz’ defined by the Global Alliance for Genomics and Health (GA4GH) Benchmarking Team (https://github.com/ga4gh/benchmarking-tools/tree/d88448a68a79ed322837bc8eb4d5a096a710993d/resources/stratification-bed-files/LowComplexity). (2) GC content intervals: ‘*_slop50.bed.gz’ defined by GA4GH Benchmarking Team (https://github.com/ga4gh/benchmarking-tools/tree/d88448a68a79ed322837bc8eb4d5a096a710993d/resources/stratification-bed-files/GCcontent). (3) coding/conserved regions: ‘refseq_uion_cds.sort.bed.gz’ defined by GA4GH Benchmarking Team (https://github.com/ga4gh/benchmarking-tools/tree/d88448a68a79ed322837bc8eb4d5a096a710993d/resources/stratification-bed-files/FunctionalRegions) were used for simulated data analysis; ‘func.37m.bed.gz’ as defined in the CHM-eval kit tool (https://github.com/lh3/CHM-eval) was used for ‘synthetic-diploid’ data analysis. (4) B allele frequency: it was calculated using AD fields in the VCF file, which records the number of reads coverage for the reference and alternative alleles. In addition, we down-sampled raw reads in real (NA12878_PrecisionFDA and NA12878_ SRR6794144) and simulated data using the tool seqtk v1.3^40^, and generated read files in 10× and 20× sequencing depth for benchmarking comparisons.

## Results

### Quality summary of WGS datasets

The two NA12878 WGS datasets, derived from PrecisionFDA and SRR6794144, had 542,906,383 and 379,033,340 read pairs, with a median insert size of 553 bp and 540 bp, and an average coverage of ~ 50× and ~ 37× (Table S1). For “synthetic-diploid” datasets, two independent replicate runs had 414,011,224 and 514,732,237 read pairs, with a median insert size of 354 bp and 329 bp, and a sequencing depth of ~ 40× and ~ 50×, respectively. About 98.7–99.4% of the sequencing reads in the real WGS data could be aligned to the reference genome (GRCh37.hs37d5). In comparison, two simulated datasets, Sim_random and Sim_user, had 390,319,108 and 390,296,059 read pairs with a sequencing depth of ~ 40×, and almost 100% of the reads could be aligned to the reference genome (Table S1). Among the datasets, the NA12878_SRR6794144 displayed an unexpectedly high level of duplicate mapped reads (26%), compared to the others (0.2–2.6%).

### Benchmarking of GATK, DRAGEN and DeepVariant variant calling pipelines

**Figure 2:**
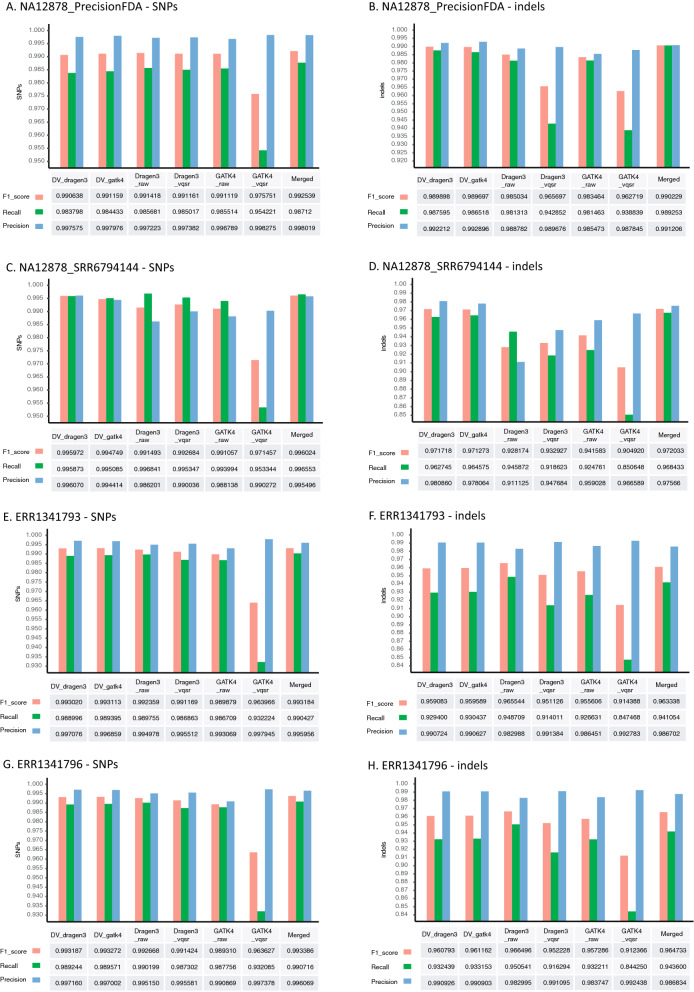
Accuracy evaluation of variant calling pipelines on real WGS datasets NA12878_PrecisionFDA (**A**,**B**), NA12878_SRR6794144 (**C**,**D**) and “synthetic-diploid” CHM1-13 (**E**,**F** for replicate ERR1341793, **G**,**H** for replicate ERR1341796). For each dataset, seven different combinations (i.e. *DV_gatk4, DV_dragen3, Dragen3_raw, Dragen3_vqsr, GATK4_raw, GATK4_vqsr* and *Merged*) were compared. The performance metrics (F1-score, Recall and Precision) of SNP and indel calls were estimated using a “genotype match” approach for NA12878 and a “local match” approach for the “synthetic-diploid” CHM1-13.

The accuracy of germline variant calls using NA12878 and “synthetic-diploid” WGS datasets was first compared. For SNP calls, all benchmarked pipelines (and their combinations) had F1-score, recall and precision values higher than 0.963, 0.932 and 0.986, respectively. Specifically, *Dragen3_raw* showed the highest F1-score value in NA12878_PrecisionFDA dataset, while *DV_Dragen3* outperformed the others in F1-score for the NA12878_SRR6794144 dataset (Fig. 2A,C). *DV_gakt4* had the best performance with respect to accuracy for the two replicate runs of “synthetic-diploid” datasets (Fig. 2E,G; Table S5). Furthermore, we found that F1-scores in five of the six combinations are close to each other, except for *GATK4_vqsr* with a range of values 0.989–0.996. The lower F1-score of *GATK4_vqsr* is mainly due to a poor performance in recall metrics, although precision metrics can reach a high value in real datasets (Fig. 2).

Compared to SNP calls, the metrics of indel calls is more diverse; F1-scores range from 0.905 to 0.989 in NA12878 dataset and from 0.912 to 0.961 in the “synthetic-diploid” dataset (Fig. 2B,D,F,H). Notably, *DV_dragen3* showed a higher F1-score than others in two datasets of NA12878, whereas the accuracy of *Dragen3_raw* gave the best performance in two replicate runs of “synthetic-diploid” (Table S5). Again, *GATK4_vqsr* suggested a poor F1-score value in all benchmarked datasets. By contrast, the benchmark evaluation on two simulated WGS datasets showed similar F1-score metrics for SNP and indel calls, respectively, in which *Dragen3_raw* was scored as having the best accuracy regardless of whether the benchmarking was done with a high confidence bed file or not (Figure S1). In total, our results indicate that the *Dragen3_raw* and *DV_dragen3* achieve better F1-scores for small variant calls in analyses of real and simulated datasets.

In order to minimize false negatives, variants called by at least two of the benchmarked pipelines (i.e. *GATK4_raw, Dragen3_raw, DV_gatk4* and *DV_*dragen3) were merged. In the real data, a minor improvement in recall metrics benefits F1-score except for indel calling in the “synthetic-diploid” datasets (Fig. 2), although a few more false positives were also introduced. In comparison, no improvement in recall values for SNPs and indels calling after variants merging was found in the simulated data (Figure S1).

### Stratification analysis of different genome contexts

We stratified the performance and evaluated benchmarking metrics in different genome contexts. Recall, precision and F1-scores that were compared in conserved and coding regions for the “synthetic-diploid” datasets were displayed in Table S2. The performances of all pipeline combinations (except *GATK4_vqsr*) were similar to each other, with F1-score ranging from 0.9944 to 0.9967 for SNP calls. Although the metrics of indel calls were variable in F1-score, differences between *DV_gatk4, DV_dragen3, Dragen3_raw* and *GATK4_raw* were not significant (Table S2). Similarly, stratification analysis on conserved/coding regions using simulated WGS datasets showed analogous F1-scores among the different pipeline combinations (Table S3).

In addition, the performance when stratified by sequence complexity, GC content, B allele frequency and sequencing depth was evaluated. As expected, the metric values (F1-score, recall and precision) of SNPs and indels tend to decrease with an increase in abundance of tandem repeats, and all pipelines gave a poor accuracy of variant calling in the low complexity regions with repeat lengths > 200 bp (Figure S2). GC content analysis on SNPs and indels showed a similar pattern, with a poor performance of F1-score in regions of high and low GC composition (Figure S3). A significant fall in precision was found at the allele fraction interval “0.1–0.2” for SNP and indel calling in stratification analysis of B allele frequencies (Figure S4). The low performance of this metric is not surprising, as it is difficult to phase genotypes and infer whether a polymorphic site is heterozygous or homozygous accurately under such allele fractions. With respect to the performance on WGS data in a gradient of read coverage, the quality of variants calling (e.g. F1-score, recall and precision) dropped with decreasing sequencing depth for all pipelines (Figure S5). At a very low depth of coverage (e.g. 10X), the DRAGEN pipeline alone (i.e. *Dragen3_raw*) or in combination with DeepVariant (i.e. *DV_dragen3*) provided a better accuracy in our comparisons, while GATK (i.e. *GATK4_raw*) was more susceptible to errors.

The analysis of substitution signatures and contexts of false positive and negative variants in the NA12878_SRR6794144 dataset demonstrated that there were more calls with A > T, C > A, G > T and T > A substitutions in *GATK4_raw* false positive variants than the expected distribution shown in the true gold callset (Figure S6A), which supports earlier findings reported by Supernat et al.^24^*.* Additionally, more C > A substitutions in both false positive and negative variants called by *Dragen3_raw* were found. In comparison, more A > C and T > G substitutions were identified in false positive variants called by *GATK4_raw* than expected in the NA12878_PrecisionFDA dataset (Figure S6B). Interestingly, a SNP type bias in the real data indicated a pipeline-specific feature. However, in the simulated data, both false positives and negatives called by all pipeline combinations seemed to be independent of compositional biases with respect to the base change (Figure S6C and D).

### Comparison of variant calling concordance

**Figure 3:**
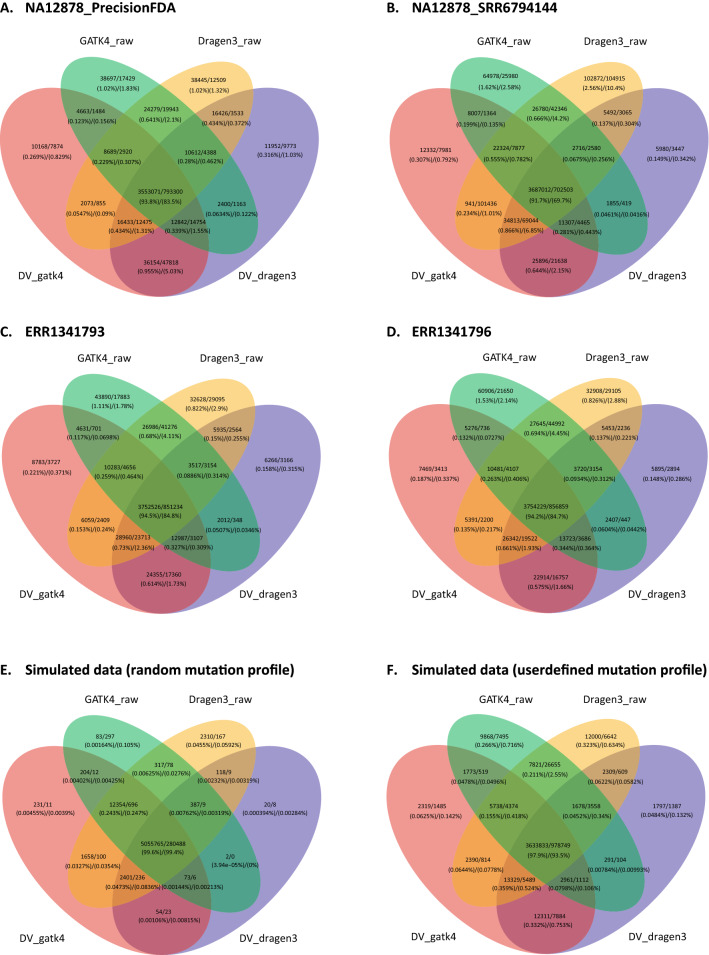
Venn diagrams showing the intersection of variants called by different pipeline combinations on NA12878_PrecisionFDA (**A**), NA12878_SRR6794144 (**B**), “synthetic-diploid” CHM1-13 (**C**—replicate ERR1341793; **D**—replicate ERR1341796) and two simulated WGS datasets (**E**—random mutation profile; **F**—user defined mutation profile). The number of SNP and indel variants are shown together using separator ‘/’. The callsets *Dragen3_vqsr* and *GATK4_vqsr* are not included in the comparison as they are subsets of *Dragen3_raw* and *GATK4_raw*, respectively.

The venn diagrams in Fig. 3 illustrate the intersection of SNPs and indels called by *GATK4_raw, Dragen3_raw, DV_gatk4* and *DV_dragen3*. For both real and simulated datasets, around 91.7–99.6% SNPs were jointly reported by all the pipeline combinations, and over 95.3–99.95% of SNPs could be detected by at least two pipeline combinations. The fractions of SNPs uniquely called by *GATK4_raw, Dragen3_raw, DV_gatk4* and *DV_dragen3* were only 0.002–1.62%, 0.045–2.56%, 0.005–0.31% and 0.0004–0.32%, respectively. By contrast, there were 83.5–99.4% indel variants commonly detected by multiple combinations in all datasets, except for NA12878_SRR6794144, in which only 69.7% of total indels were jointly identified (Fig. 3). Although indels have a larger divergence of calling concordance compared to SNPs, the high number of variants detected by multiple combinations and low orphan variants support a good agreement in the identification of SNPs and indels by different pipelines.

### Comparison of execution time

**Figure 4:**
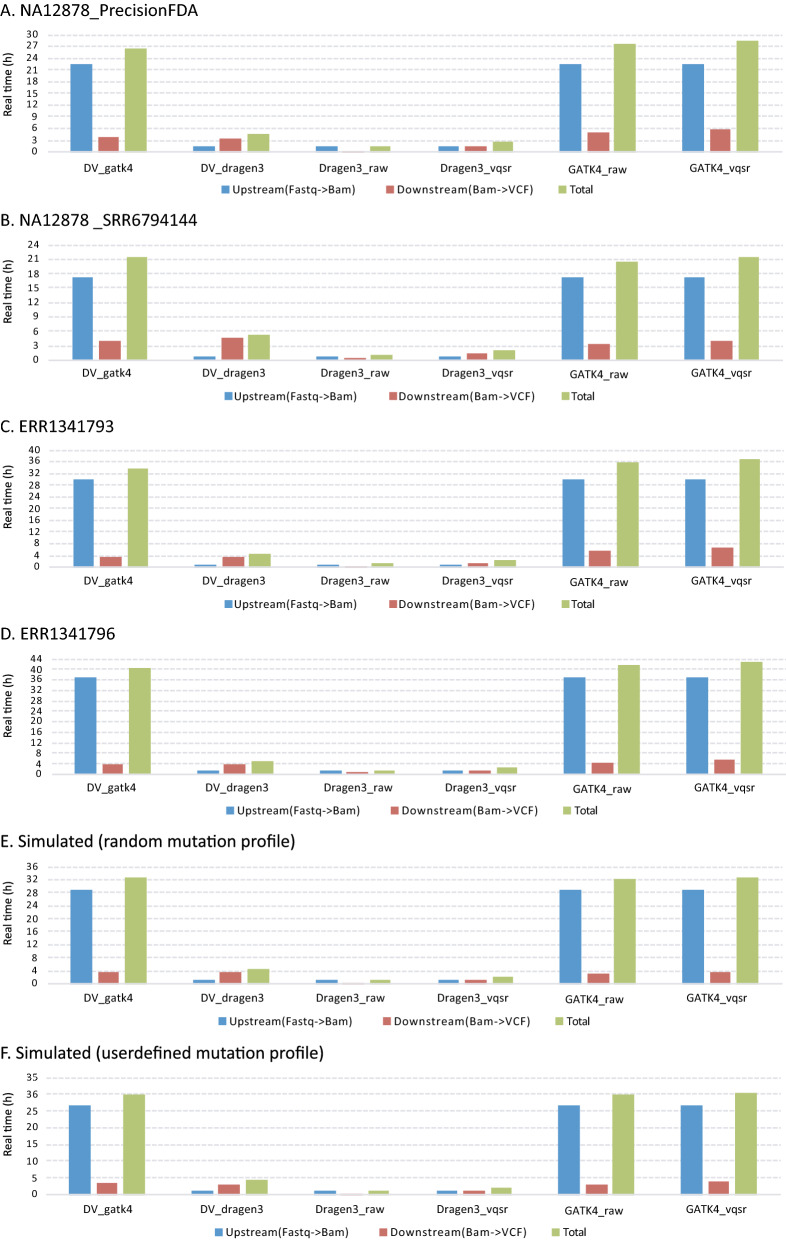
Variant calling runtime of six pipeline combinations (*DV_gatk4, DV_dragen3, Dragen3_raw, Dragen3_vqsr, GATK4_raw* and *GATK4_vqsr*) benchmarked on a HPC cluster (**A**,**B**—NA12878_PrecisionFDA and NA12878_SRR6794144 datasets; **C**,**D**—“synthetic-diploid” ERR1341793 and ERR1341796 datasets; **E**,**F**—simulated data based on a random and a user defined mutation profile).

To better assess the operating efficiency, the pipeline processing procedure was divided into upstream (Fastq to BAM file) and downstream (BAM to VCF file) workflows, and the runtime of each workflow was measured. For benchmarking execution time on a HPC cluster, *Dragen3_raw/vqsr* took from 30 min to 1.5 h in the upstream analysis. This was significantly lower than *GATK4_raw/vqsr,* with a speed-up gain in the range of 17× to 33× (Fig. 4). In the downstream workflow, *Dragen3_raw/vqsr* still outperformed *GATK4_raw/vqsr* and *DV_gatk4/DV_dragen3,* despite the degree of speed-up gain being lower than that of the upstream workflow. Similarly, DRAGEN showed a big advantage in running speed when compared on a local VM, with a time requirement of even less than that of benchmarked on the HPC cluster (Figure S7). Overall, compared to the other pipelines, DRAGEN platform provided an ultra-rapid analysis solution for germline variant calling using WGS data.

## Discussion

In this study, we empirically evaluated the performance of different pipelines (and their combinations) for germline variant calling using real and simulated WGS data. Our results demonstrated that DeepVariant (*DV_dragen3* or *DV_gatk4*) shows a higher accuracy in SNP calls for one NA12878 dataset (SRR6794144) and two “synthetic-diploid” datasets, and in indel calls for two NA12878 datasets. Despite a better performance, the F1-scores obtained in NA12878 benchmarking evaluation were lower than those published in the FDA Truth Challenge: 0.9912–0.9959 versus 0.9996 (pFDA top) for SNP calls, and 0.9897–0.9717 versus 0.9934 (pFDA top) for indel calls. This variation probably results from differences in the benchmarking procedure of pFDA Truth challenge, in which the NA12878 sample was used for training, and the HG002 sample was used for testing. The top benchmarking results in pFDA Truth challenge were derived from the HG002 comparison. The accuracy of the DRAGEN pipeline (*Dragen3_raw*) gave a better performance in both SNP and indel calls for the simulated dataset, and in indel calls for the “synthetic-diploid” datasets, despite not achieving as high F1-score metrics as DeepVariant in the benchmark of the NA12878 dataset. In fact, the differences in benchmarking scores between DRAGEN and DeepVariant are quite small (Fig. 2 and Figure S1). In particular, stratification analysis of conserved and coding regions suggests nearly the same accuracy between them. Thus, merging variants called by multiple pipelines can reduce false negatives in a benchmarking study, which potentially benefits the F1-score. However false positives will be introduced by this, in particular for incongruent genotypes phased by different callers. In terms of a tradeoff relationship between recall and precision, the F1-score does not always indicate an improvement (it depends on the ratio between reduction of false negatives and gain of false positives).

The most important advantage of the DRAGEN platform is computational time, and consequently throughput of the massive volumes of data. Indeed, in this study the running efficiency of the DRAGEN platform was far superior to both GATK and DeepVariant with the support of hardware-based accelerations. Based on these considerations, and the accuracy results we measured, it seems reasonable to recommend that either the DRAGEN pipeline is used alone (*Dragen3_raw*), or in combination (*DV_Dragen3*), where DRAGEN is used for upstream processing and DeepVariant for downstream processing, to obtain a balance in accuracy and efficiency for germline variant calling from WGS data.

Although the DRAGEN platform provided the best performance in running efficiency in this study, real execution time on HPC clusters never reached the performance stated by the manufacturer. Even when a benchmarking comparison was performed on a local VM, where faster I/O communication on the local ext4 file system could benefit the running speed compared to a BeeGFS network file system on a HPC cluster, only minor improvements in time consumption could be observed (Figure S5). Thus, there is still room for optimizing runtime of DRAGEN platform with regards to its implementation at the infrastructure and hardware levels. Compared to DRAGEN, optimization of running efficiency for GATK and DeepVariant was not achieved in the computing environment of our study. For example, DeepVariant could gain a 2.5× speedup using a high-performance graphics processing unit, since its variant calling algorithm is based on image analyses. For GATK, the genome was split into 14 fractions by chromosomes, scaffolds and contigs, and were run in a “scatter–gather” strategy. There were 64 cores per node in the HPC cluster, therefore the genome could ideally be split into the same number of divisions as the number of cores, and be run in parallel. Despite these optimizations, neither DeepVariant nor GATK would achieve the efficiency of DRAGEN, as no hardware-accelerated implementations of genomic analyses algorithms have been developed for them.

Two types of high confidence benchmark truth call sets: the GiaB reference data (sample NA12878) and the “synthetic-diploid” mixture of two haploid cell lines were applied to evaluate the performance of germline variant callers using real data. The construction of the truth set, and strengths and weaknesses based on variant type and genome context should be considered. The GiaB benchmark sets were built from the consensus of multiple variant callers on Illumina short-read sequencing with the aid of a pedigree analysis, integration of structural variants identified with long fragment technologies by PacBio and 10X Genomics, and HuRef genome analysis using Sanger sequencing^39^. Nearly all the “true” variants in NA12878 sample are present in the resource files (e.g. dbSNP, 1000 genomes and the training data for DeepVariant) used for pipeline running. In this case, the results are likely overfitting as the answer has been used all along. Furthermore, truth callset of NA12878 excludes more difficult types of variants in the region with moderately diverged repeats, and segmental duplications, as consensus in such regions has not been reached. This will tend to bias GiaB datasets towards “easy-to-sequence-and-analyze” genome regions.

The truth ”synthetic-diploid” callset was generated by assembly of long reads sequenced from two haploid cell lines (CHM1 and CHM13) using PacBio technology. This can be considered trustworthy, as there are no heterozygous sites that tend to confuse the assembly. The exclusive use of PacBio, without incorporation of the flaws generated from Illumina’s short-read technology, ensure there is less correlation between the failure modes of this method on the short-read data and confidence regions. This enables benchmarking in regions that are difficult to map with short reads. However, the “synthetic-diploid” callset currently contains some errors that were intrinsically present in the long reads^27^. It is thus recommended to use a less strict benchmarking strategy (“local matches” method) for comparisons^27,39^. Here, the evaluation using “genotype match” as it applied in NA12878 datasets was also performed (Table S4). For SNP metrics, DeepVariant (*DV_gatk4 or DV_dragen3)* was consistently rated as the best according to their respective F1-scores. In terms of the performance metrics of indel calls, *Dragen3_raw* and *GATK4_raw* had a better value for the ERR1341793 and ERR1341796 datasets, respectively. As expected, the recall, precision and F1-score of indels are relatively low compared to the metrics done by the “local match” method. Precisely assessing the accuracy of genotypes from the exact sequence changes in REF and ALT fields of the VCF file for “synthetic-diploid” data benchmarking remains challenging. Consequently, a less stringent methodology, like the “local match” approach is required. One advantage is robust towards representational differences of variants in truth and inquiry sets. Overall, the characteristics of these two truth datasets make them very valuable for performing a comprehensive comparison assessment of different bioinformatics tools.

In addition to the real WGS data, we generated two simulated WGS datasets on the basis of a random and a user defined mutation profile. One advantage of using simulated in silico data for benchmarking is that all “true” positive SNPs and indels are known, without the presence of controversial genotypes. The calculation of F1-score is more accurate, due to the reduced risk of overestimating false negatives. Additionally, in simulated data, the read coverage across the whole genome region has a more even distribution than that found in real data, so variant calling errors arising from low coverage in some regions could be reduced. On the other hand, the accuracy of variant calling in simulated data easily reaches saturation (Figure S1), as simulated data can achieve a perfect alignment (almost 100%, Table S1) to the reference genome, which benefits variant calling for both SNPs and indels. Furthermore, a difference in stratification analysis of GC content was found between in silico and real data, with less divergent performance metrics shown in simulated data (Figure S3). Similarly, both false positive and negative variants called by benchmarked pipelines in simulated data are independent of any types of SNP biases in the distribution of substitution signature (Figure S6). All these systematic discrepancies between simulated and real data suggest in silico data cannot capture true experimental variability and are always less complex than the real data^41,42^. Specifically, the models used for data simulation may not replicate an identical sequence complexity in real data with regard to all biological and technological features. For examples, some important modelling parameters, such as PCR amplification during library preparation, GC% coverage bias, sequencing errors and mutation profile were empirically learned from selected known datasets without considering sample specificity and diversity broadly. As the results showed, the benchmarked pipelines can identify most true positives well, without introducing variable false positives when variants calling is carried out on simulated reads. Although the models do not fit a real scenario completely, simulation is still an important approach for benchmarking evaluation of different bioinformatics pipelines with similar functionality. However, it should be noted that the application of simulated data in benchmarking can only complement the real experimental gold standard data, as a useful supplement for testing and development of computational tools. In silico data do not replace the use of physical standards that measure the full range of variation as faced in clinical diagnostics^42^.

It is highly recommended in GATK and DRAGEN best practices to apply variant quality score recalibration (VQSR) to filter raw SNP and indel calls generated by HaplotypeCaller, and to remove calling artefacts. In theory, VQSR balances sensitivity and specificity during variant filtering. However, the F1-score was lower in both real and simulated data except for *Dragen3_vqsr* in NA12878_SRR679414 after VQSR filtering, although precision reached the highest value. In Fig. 2, the precision metrics on average were raised only 0.15% and 0.5% for SNPs and indels, respectively, while the recall suffered from a larger fall, which is significant for *GATK4_vqsr* (e.g. reduced by 3% for SNPs and 4% for indels in NA12878_PrecisionFDA dataset). Consequently, the calculated F1-score did not show the expected improvement. This could potentially be explained by the fact that VQSR was performed on a single sample at a time, yielding instability from the convergence failure of core algorithm modelling. This may lead to the necessity for quite “strict” criteria in the filtering of raw variant calls and cause a lower recall value. In addition, we experienced some challenges in performing VQSR analysis on the simulated WGS data under the default parameters, as there were not enough variants to be trained as a meaningful “bad set”, for effective cluster discrimination. Instead, we turned down the number of max-gaussian parameters to 2 for indels and 4 for SNPs and forced the program to group variants into a smaller number of clusters to satisfy the statistical requirements. Overall, our results suggest it is not necessary to perform VQSR control for one sample analysis, and in fact the raw unfiltered VCF files have a good balance between recall and precision for GATK and DRAGEN.

Several caveats and limitations of the current study needs mention. First, variant calling was performed by the pipelines using their default parameters. It would be interesting to attempt to optimize the parameters and settings for each pipeline, potentially benefitting the variant calling accuracy. However, this is in general a time-consuming process sometimes requiring communication with the authors of each tool for a deep investigation of parameter usage. Second, we performed a benchmarking study using both real and simulated data. A further technique is to design ‘semi-simulated’ datasets that combine real experimental data with an in silico (i.e. computational) spike signal. For example, by combining cells from ‘null’ (e.g. healthy) samples with a subset of cells from samples expected to contain a true differential signal. This strategy can create datasets with more realistic levels of variability and correlation, together with a ground truth. Lastly, we did not include all available germline variants calling pipelines for benchmarking study, and three of them (i.e. GATK, DRAGEN and DeepVariant) were chosen for this study, although others with similar functionality exist (e.g. Strelka2). We focused on these three because they represented the most up-to-date and widely used tools for germline variant calling using WGS data. Recently, the GATK team announced a collaboration with the Illumina DRAGEN team to co-develop analysis methods and pipelines for short-read variant calling. DRAGEN-GATK is likely to be released in the near future, which appears to be able to provide researchers with tools that are fast, reproducible and accurate under an open-source framework, and should deserve attention in further studies.

In conclusion, our benchmarking on real and simulated WGS datasets reveal DRAGEN and DeepVariant pipelines have high accuracy in small germline variant calling, and there are no significant differences in their F1-score performances. The DRAGEN platform performed superiorly in ultra-rapid analysis of WGS data for SNP and indel detection, and therefore has great potential for implementation in routine genomic medicine, where speed may be of essence. The combination of DeepVariant and DRAGEN pipelines can also offer a fast, efficient and reliable way to analyze WGS data on a large scale, and go a long way toward reliable and consistent calling of variants when translating genetic variant information to medical diagnostics.

## Supplementary information

Supplementary Figures.

Supplementary Tables.

## Data Availability

Raw WGS data of NA12878 (HG001) were publicly obtained from https://precision.fda.gov/challenges/truth and NCBI SRA repository (https://trace.ncbi.nlm.nih.gov/Traces/sra/?run=SRR6794144), respectively. Raw reads of “Synthetic-diploid” WGS data sequenced from mixed DNA of CHM1 and CHM13 cell lines were retrieved from the European Nucleotide Archive repository (https://www.ebi.ac.uk/ena/data/view/SAMEA3911976). Raw reads of simulated WGS data generated and analyzed in this study are available from the corresponding authors (E.H) on request. The scripts used for running variant calling pipelines are available on the GitHub page: https://github.com/senzhaocode/Benchmark_script
