# Supplementary material for: Accuracy and efficiency of germline variant calling pipelines for human genome data
Source: Sci Rep. 2020 Nov 19;10:20222. doi: 10.1038/s41598-020-77218-4 (PMC7678823; doi:10.1038/s41598-020-77218-4)
Supplement: Supplementary file 1 — Supplementary Figures. [file 41598_2020_77218_MOESM1_ESM.pdf]

# Supplementary Figures

## Accuracy and efficiency of germline variant calling pipelines for human genome data

Sen Zhao[1], Oleg Agafonov[2], Abdulrahman Azab[3,4], Tomasz Stokowy[5,6], Eivind Hovig[1,3*]

1 Department of Tumor Biology, Institute of Cancer Research, The Norwegian Radium Hospital, Oslo University Hospital, 0310 Oslo, Norway
2 DNV GL, 1363 Høvik, Norway
3 Center for bioinformatics, Department of Informatics, University of Oslo, 0316 Oslo, Norway
4 Research Support Services Group, University Center for Information Technology, 0316 Oslo, Norway
5 Computational Biology Unit, Institute of Informatics, University of Bergen, 5008 Bergen, Norway
6 Department of Clinical Science, University of Bergen, 5021 Bergen, Norway

* To whom correspondence should be addressed.
Tel: (+47)93069881
Email: ehovig@ifi.uio.no

A. Simulated (random mutation profile) - SNPs

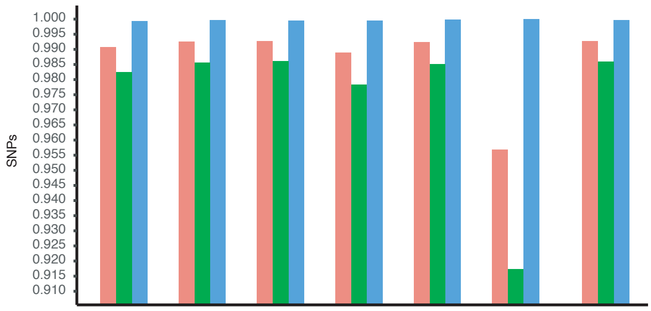


| | DV_dragen3 | DV_gatk4 | Dragen3 _raw | Dragen3 _vqsr | GATK4 _raw | GATK4 _vqsr | Merged |
|---|---|---|---|---|---|---|---|
| F1_score | 0.990803 | 0.992578 | 0.992786 | 0.988857 | 0.992401 | 0.956906 | 0.99272 |
| Recall | 0.982421 | 0.985613 | 0.986086 | 0.978365 | 0.985109 | 0.917378 | 0.985812 |
| Precision | 0.999330 | 0.999643 | 0.999579 | 0.999576 | 0.999802 | 0.999993 | 0.999725 |

B. Simulated (random mutation profile) - indels

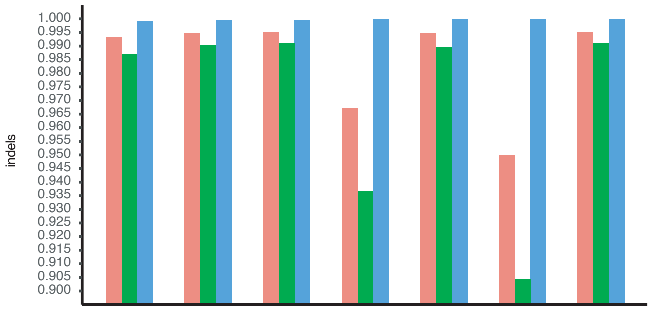


| | DV_dragen3 | DV_gatk4 | Dragen3 _raw | Dragen3 _vqsr | GATK4 _raw | GATK4 _vqsr | Merged |
|---|---|---|---|---|---|---|---|
| F1_score | 0.993141 | 0.994948 | 0.995262 | 0.967213 | 0.994646 | 0.949805 | 0.995131 |
| Recall | 0.987064 | 0.980329 | 0.991032 | 0.936558 | 0.989553 | 0.904424 | 0.990663 |
| Precision | 0.999293 | 0.999610 | 0.999529 | 0.999944 | 0.999791 | 0.999981 | 0.999639 |

C. Simulated (userdefined mutation profile) - SNPs

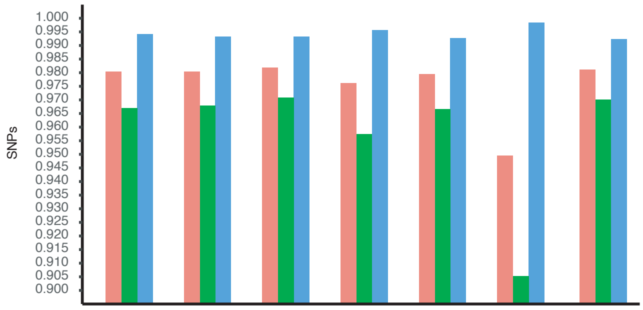


| | DV_dragen3 | DV_gatk4 | Dragen3 _raw | Dragen3 _vqsr | GATK4 _raw | GATK4 _vqsr | Merged |
|---|---|---|---|---|---|---|---|
| F1_score | 0.980336 | 0.980320 | 0.981865 | 0.976108 | 0.979361 | 0.949287 | 0.981083 |
| Recall | 0.966984 | 0.967792 | 0.970702 | 0.957356 | 0.966482 | 0.905098 | 0.970033 |
| Precision | 0.994062 | 0.993177 | 0.993289 | 0.995611 | 0.992588 | 0.998013 | 0.992387 |

D. Simulated (userdefined mutation profile) - indels

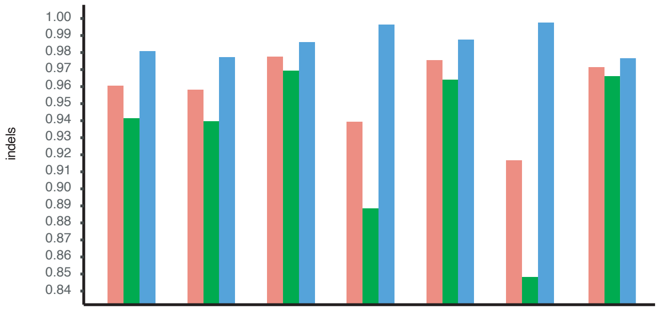


| | DV_dragen3 | DV_gatk4 | Dragen3 _raw | Dragen3 _vqsr | GATK4 _raw | GATK4 _vqsr | Merged |
|---|---|---|---|---|---|---|---|
| F1_score | 0.960696 | 0.958139 | 0.977657 | 0.939439 | 0.975587 | 0.915420 | 0.9705 |
| Recall | 0.941496 | 0.939597 | 0.969263 | 0.888650 | 0.963996 | 0.847836 | 0.964327 |
| Precision | 0.980696 | 0.977428 | 0.986197 | 0.996385 | 0.987460 | 0.994711 | 0.976752 |

E. Simulated (random mutation profile) - SNPs

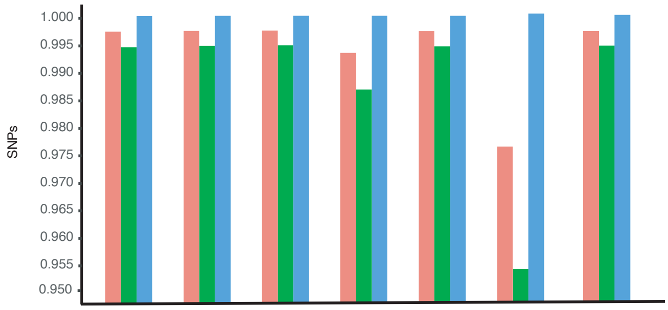


| | DV_dragen3 | DV_gatk4 | Dragen3 _raw | Dragen3 _vqsr | GATK4 _raw | GATK4 _vqsr | Merged |
|---|---|---|---|---|---|---|---|
| F1_score | 0.997224 | 0.997089 | 0.997290 | 0.993231 | 0.997193 | 0.976691 | 0.997275 |
| Recall | 0.994495 | 0.994258 | 0.994618 | 0.986578 | 0.994429 | 0.954448 | 0.99459 |
| Precision | 0.999968 | 0.999935 | 0.999975 | 0.999975 | 0.999973 | 0.999996 | 0.999975 |

F. Simulated (random mutation profile) - indels

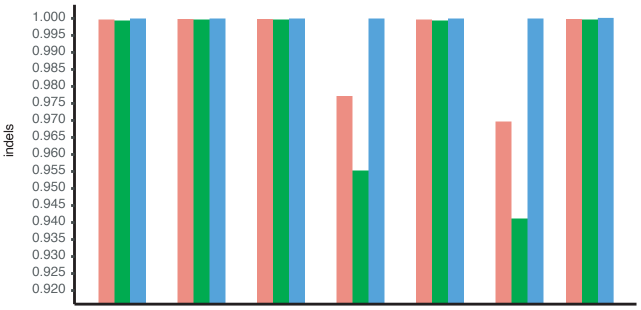


| | DV_dragen3 | DV_gatk4 | Dragen3 _raw | Dragen3 _vqsr | GATK4 _raw | GATK4 _vqsr | Merged |
|---|---|---|---|---|---|---|---|
| F1_score | 0.999793 | 0.999626 | 0.999836 | 0.977101 | 0.999688 | 0.969658 | 0.999811 |
| Recall | 0.999622 | 0.999322 | 0.999712 | 0.955231 | 0.999392 | 0.941118 | 0.999684 |
| Precision | 0.999965 | 0.999930 | 0.999961 | 0.999996 | 0.999984 | 0.999983 | 0.999938 |

G. Simulated (userdefined mutation profile) - SNPs

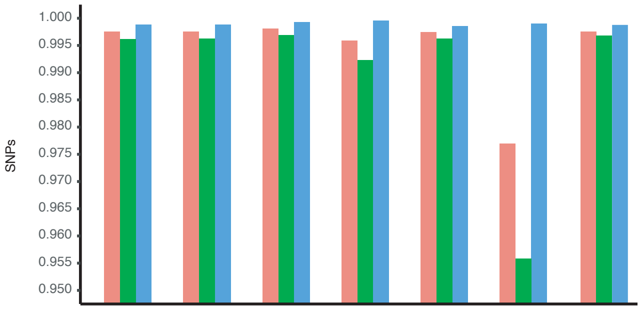


| | DV_dragen3 | DV_gatk4 | Dragen3 _raw | Dragen3 _vqsr | GATK4 _raw | GATK4 _vqsr | Merged |
|---|---|---|---|---|---|---|---|
| F1_score | 0.997502 | 0.997477 | 0.998048 | 0.995880 | 0.997395 | 0.976920 | 0.997668 |
| Recall | 0.996194 | 0.996109 | 0.996853 | 0.992246 | 0.996226 | 0.955811 | 0.996661 |
| Precision | 0.998814 | 0.998849 | 0.999245 | 0.999541 | 0.998566 | 0.998983 | 0.998677 |

H. Simulated (userdefined mutation profile) - indels

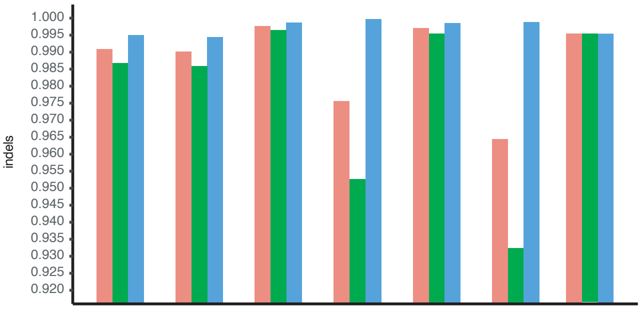


| | DV_dragen3 | DV_gatk4 | Dragen3 _raw | Dragen3 _vqsr | GATK4 _raw | GATK4 _vqsr | Merged |
|---|---|---|---|---|---|---|---|
| F1_score | 0.990099 | 0.990898 | 0.997584 | 0.975621 | 0.997001 | 0.964460 | 0.994633 |
| Recall | 0.985867 | 0.986770 | 0.996433 | 0.952716 | 0.995449 | 0.932326 | 0,99477 |
| Precision | 0.994368 | 0.995060 | 0.998739 | 0.999654 | 0.998557 | 0.998889 | 0.994496 |

**Figure S1.** Accuracy evaluation of variant calling pipelines on two simulated WGS datasets generated using random mutation profile (A and B - without using high conf. region bed file; E and F - using high conf. region bed file) or userdefined mutation profile (C and D - without using high conf. region bed file; G and H - using high conf. region bed file), respectively. For each dataset, seven different combinations (DV_gatk4, DV_dragen3, Dragen3_raw, Dragen3_vqsr, GATK4_raw, GATK4_vqsr and Merged) were compared. The performance metrics (F1-score, Recall and Precision) of SNP and indel calls were estimated using "genotype match" approach.

**Figure S2A.** Stratification analysis of SNP calling in low complexity regions (benchmarked without using high conf. regions). X-axis labels "<51bp", "51-200bp" and ">200bp" represent a total length of repeat less than 51bp, from 51 to 200 bp and larger than 200 bp.

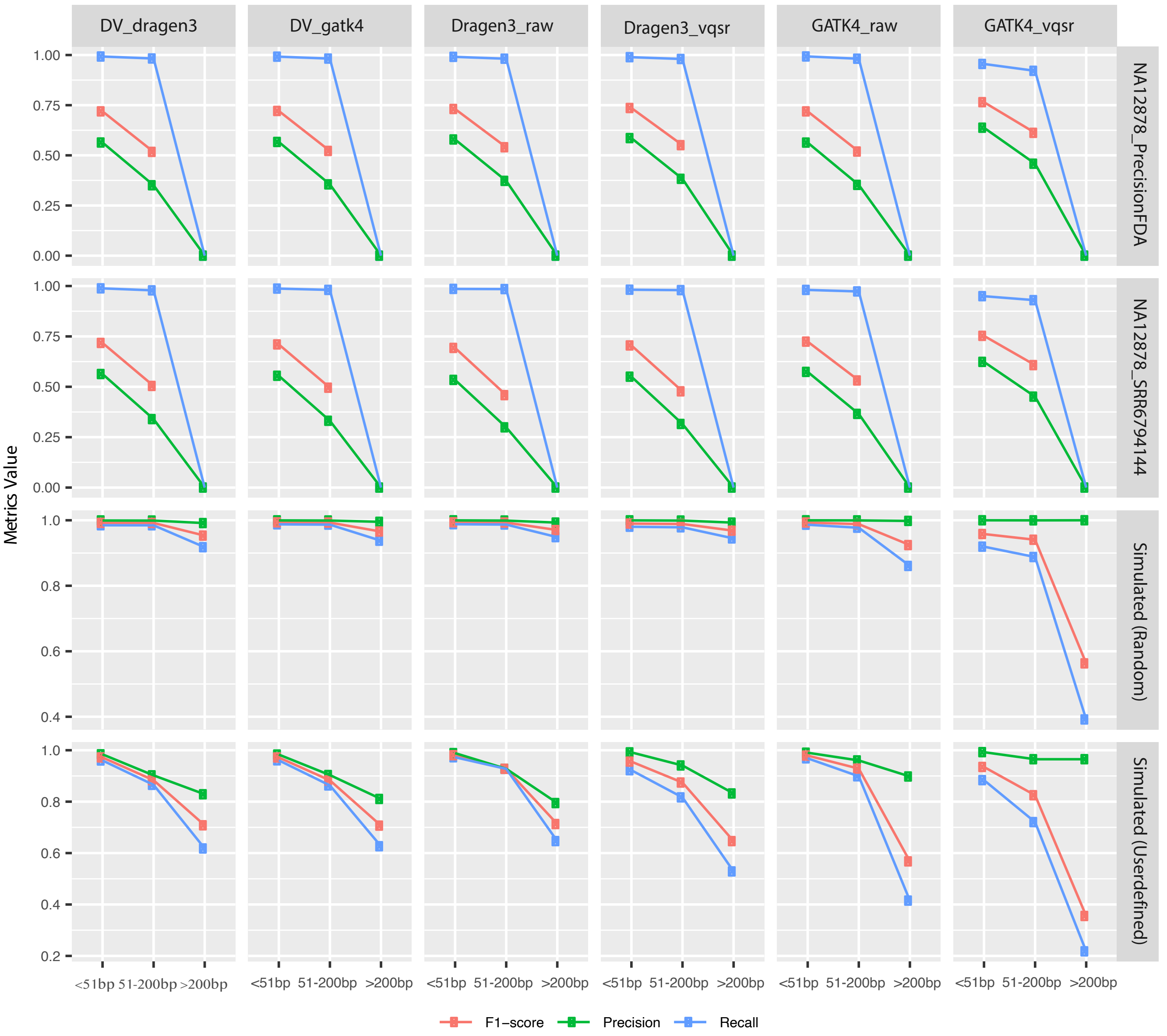

**Figure S2B.** Stratification analysis of indel calling in low complexity regions (benchmarked without using high conf. regions). X-axis labels “<51bp”, “51-200bp” and “>200bp” represent a total length of repeat less than 51bp, from 51 to 200 bp and larger than 200 bp.

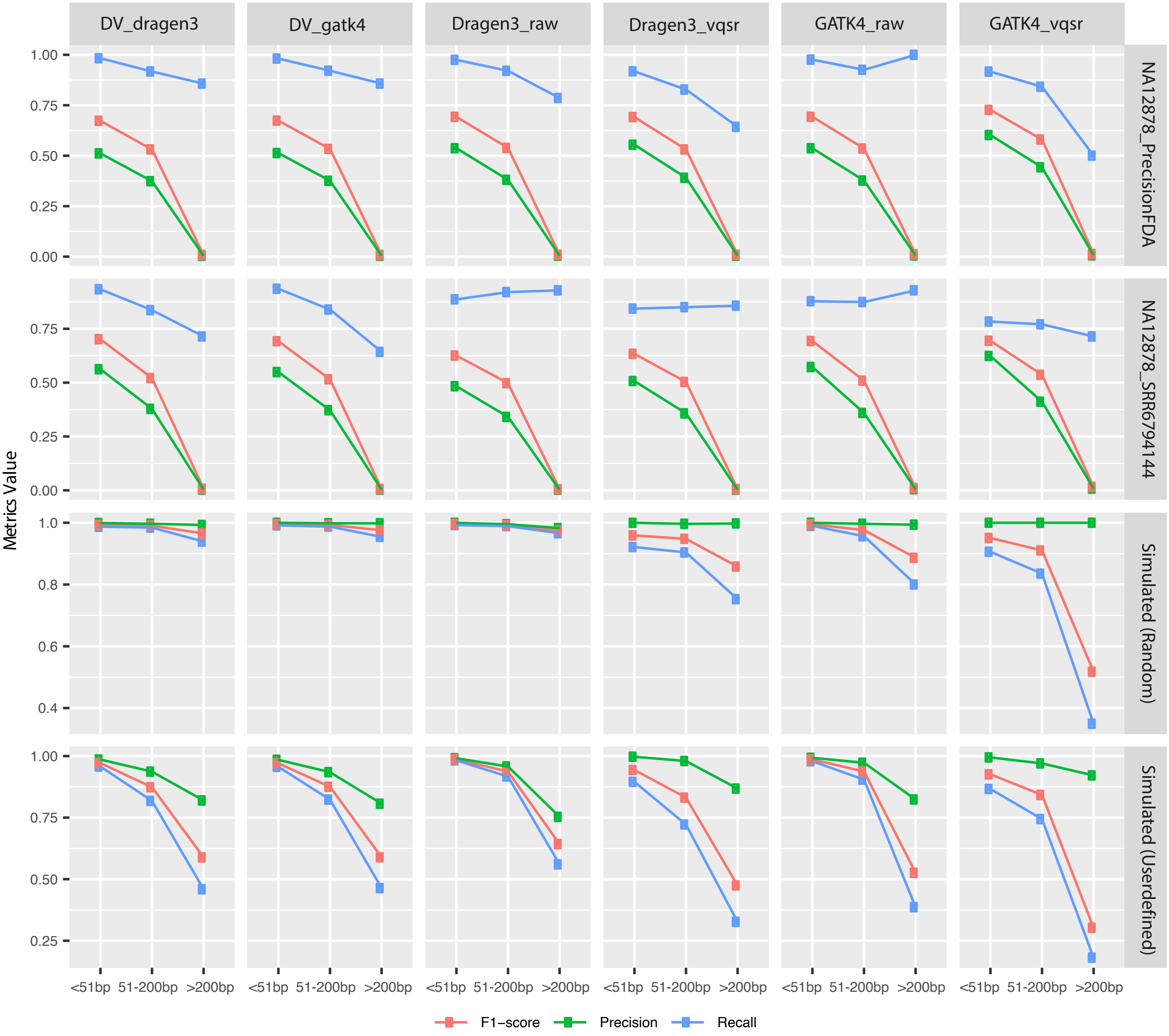

**Figure S3A.** Stratification analysis of SNP calling in different GC content regions (benchmarked without using high conf. regions)

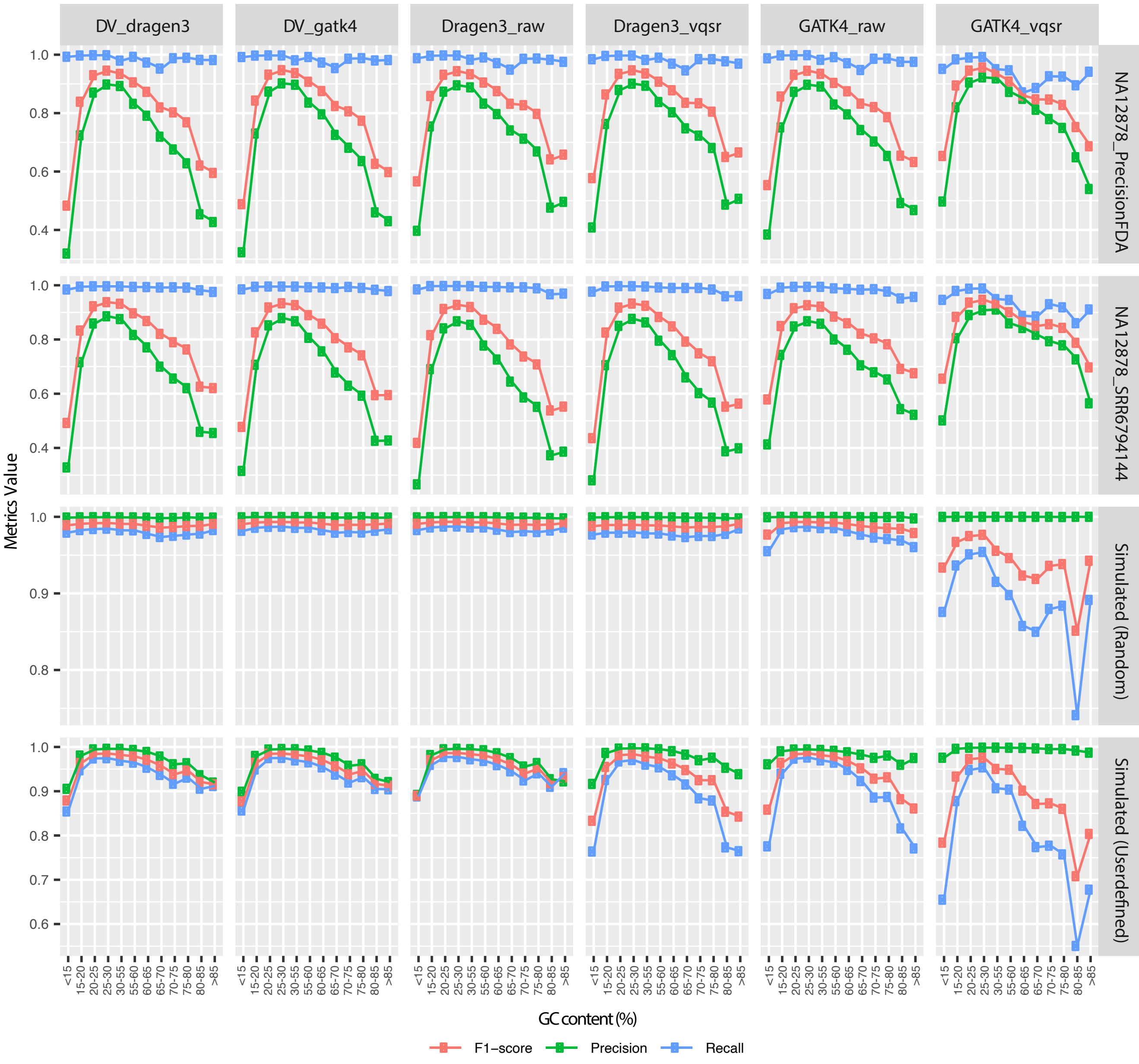

**Figure S3B.** Stratification analysis of indel calling in different GC content regions (benchmarked without using high conf. regions)

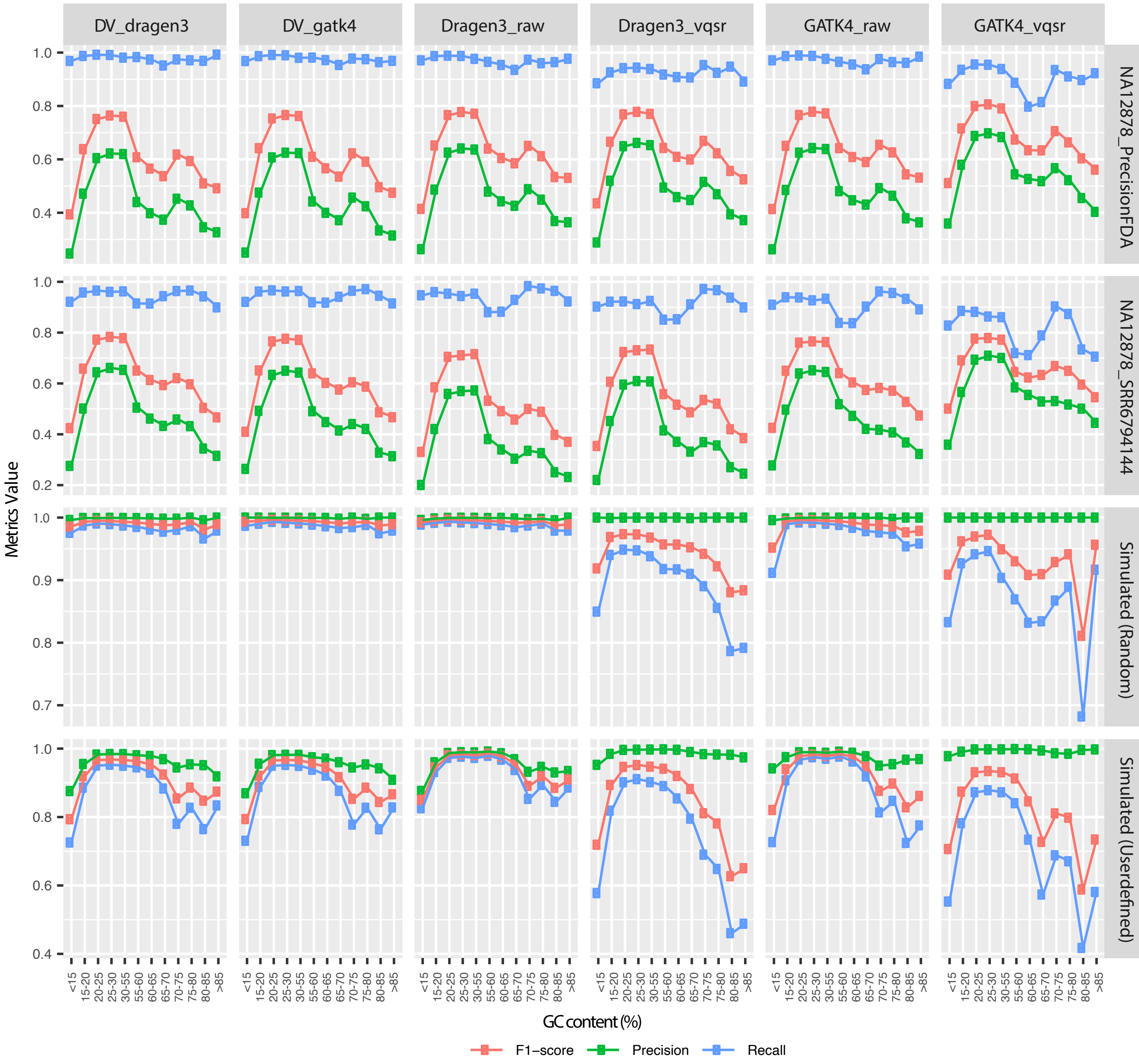

**Figure S4A.** Stratification analysis of SNP calling with different B allele frequency (for NA12878_PrecisionFDA and NA12878_SRR6794144 - benchmarking done with high conf. regions; for Simulated_Random and Simulated_Userdefined - benchmarking done without high conf. regions)

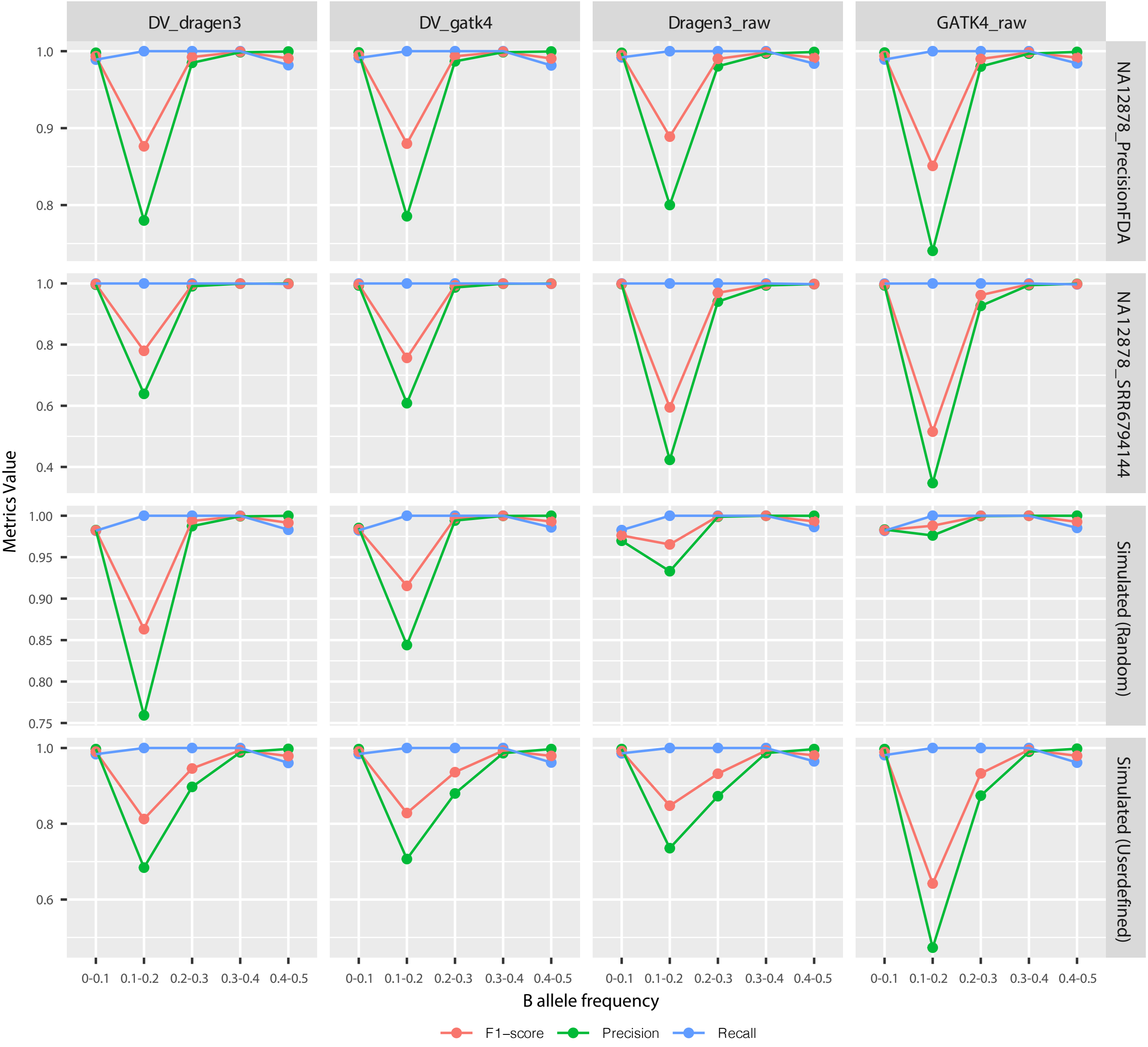

**Figure S4B.** Stratification analysis of indel calling with different B allele frequency (for NA12878_PrecisionFDA and NA12878_SRR6794144 - benchmarking done with high conf. regions; for Simulated_Random and Simulated_Userdefined - benchmarking done without high conf. regions)

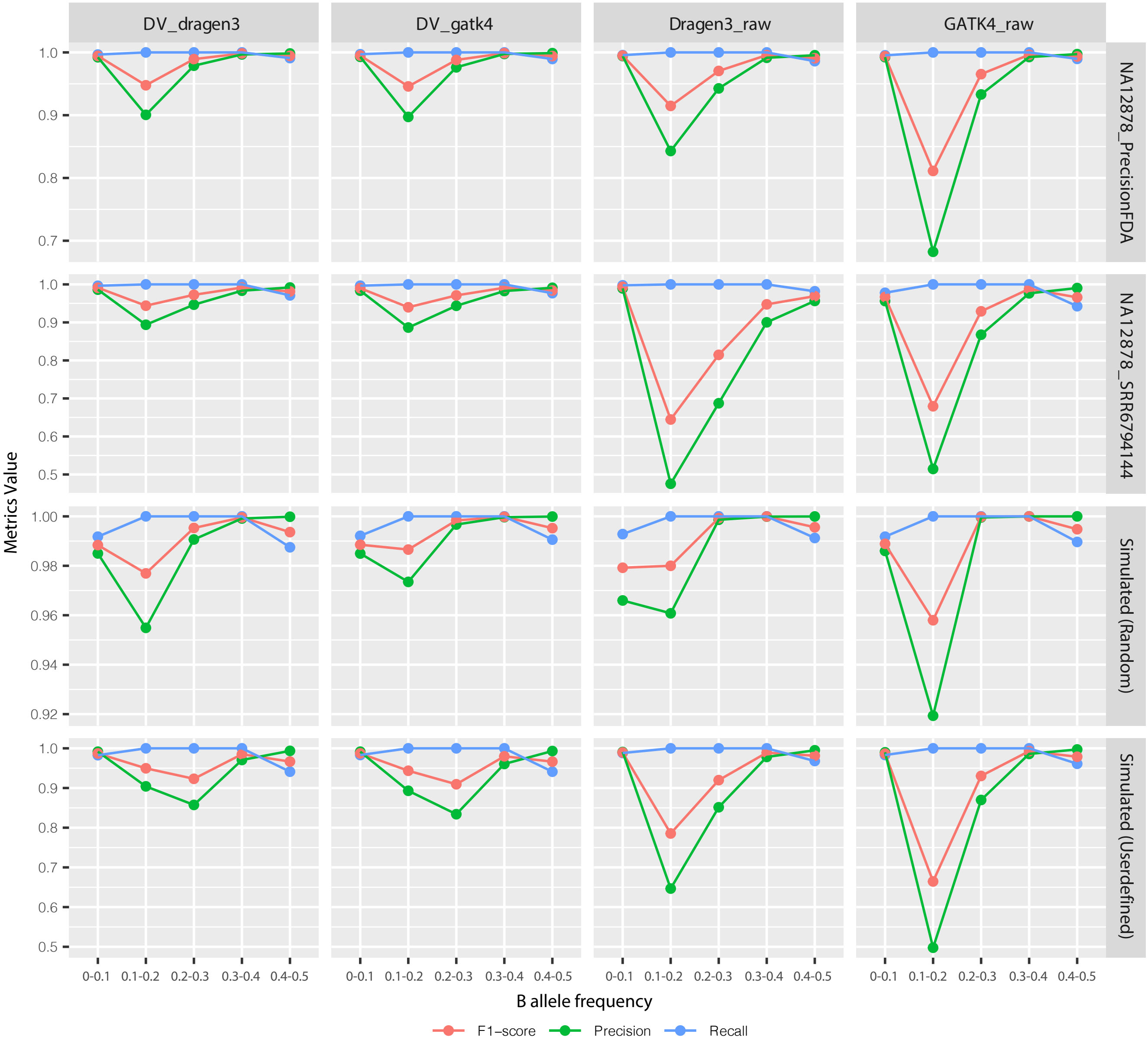

**Figure S5A.** Benchmarking analysis of SNP calling at 10X and 20X sequencing coverage (for NA12878_PrecisionFDA and NA12878_SRR6794144 - benchmarking done with high conf. regions; for Simulated_Random and Simulat-ed_Userdefined - benchmarking done without high conf. regions)

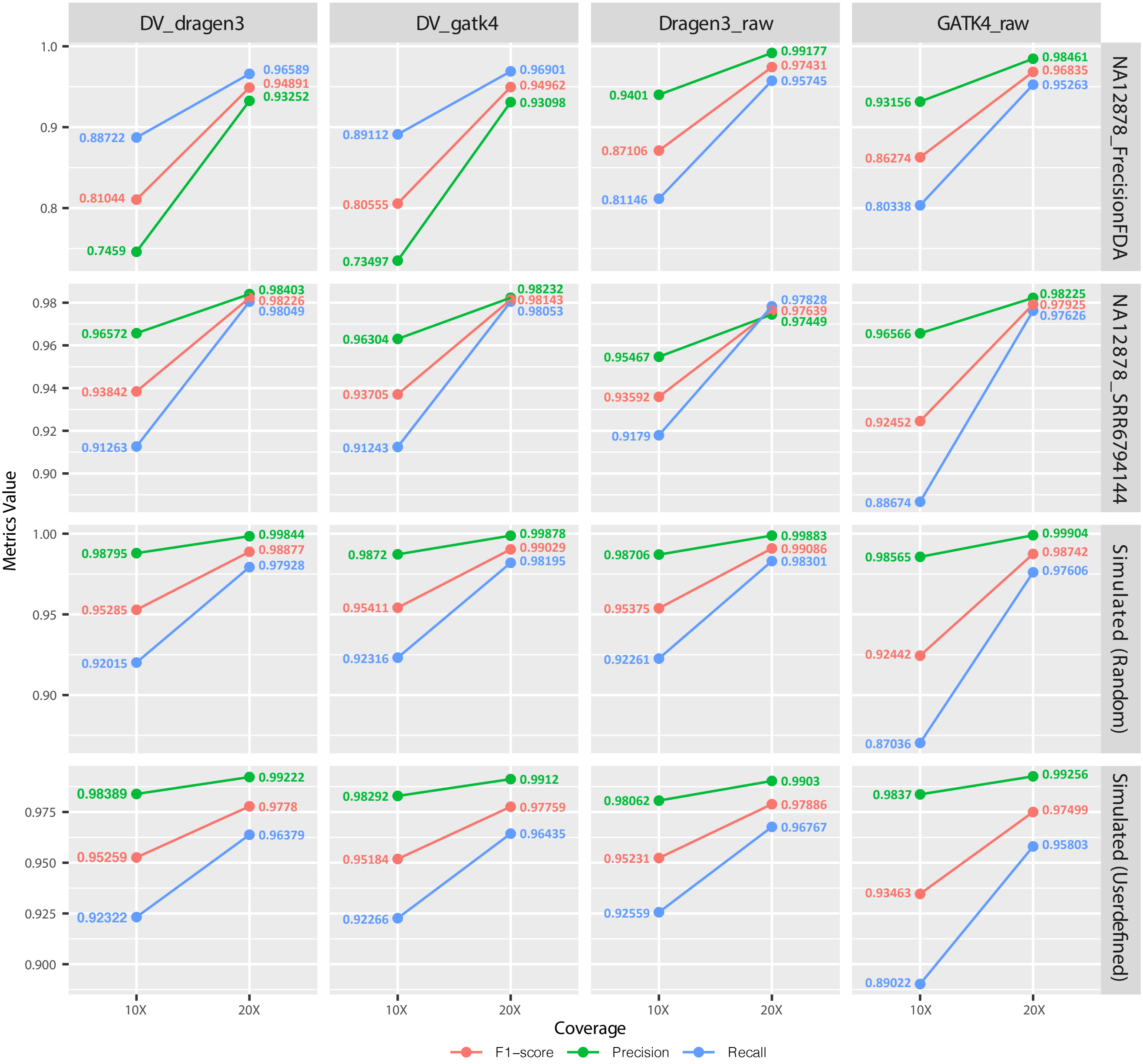

**Figure S5B.** Benchmarking analysis of indel calling at 10X and 20X sequencing coverage (for NA12878_PrecisionFDA and NA12878_SRR6794144 - benchmarking done with high conf. regions; for Simulated_Random and Simulat-ed_Userdefined - benchmarking done without high conf. regions)

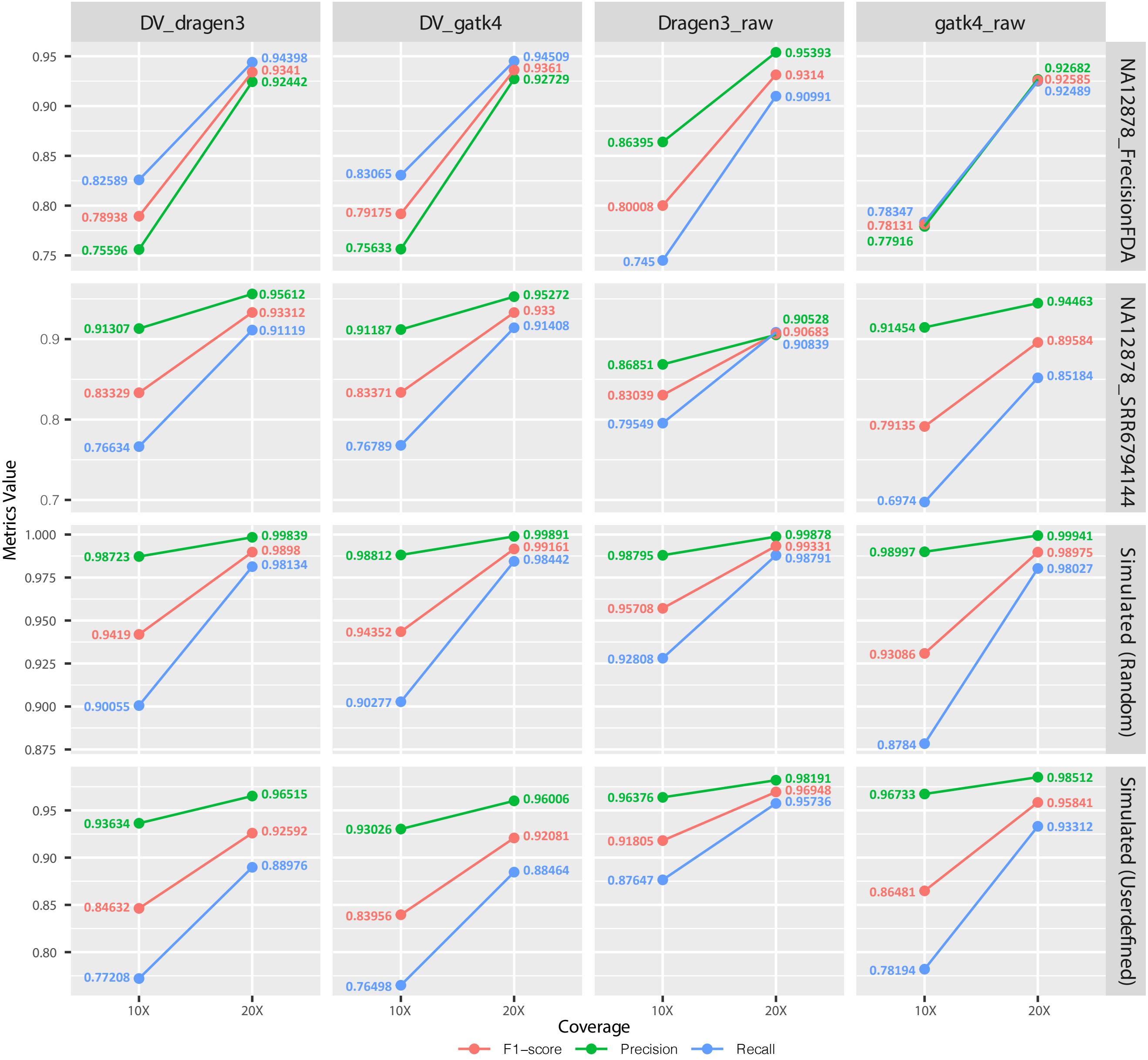

**Figure S6A.** The distribution of substitution signature of false positive and negative variants for NA12878_SRR6794144 dataset

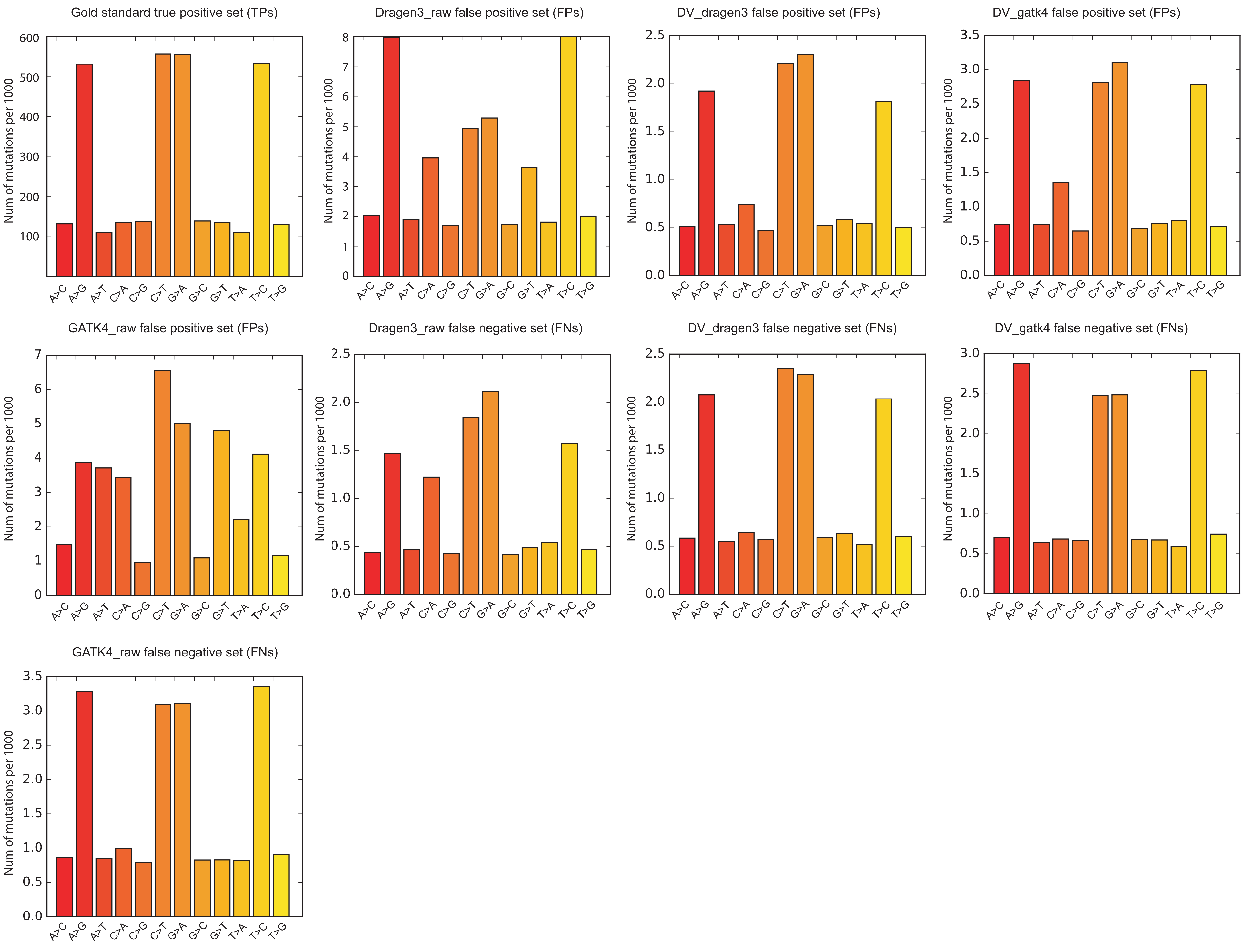

**Figure S6B.** The distribution of substitution signature of false positive and negative variants for NA12878_PrecisionFDA dataset

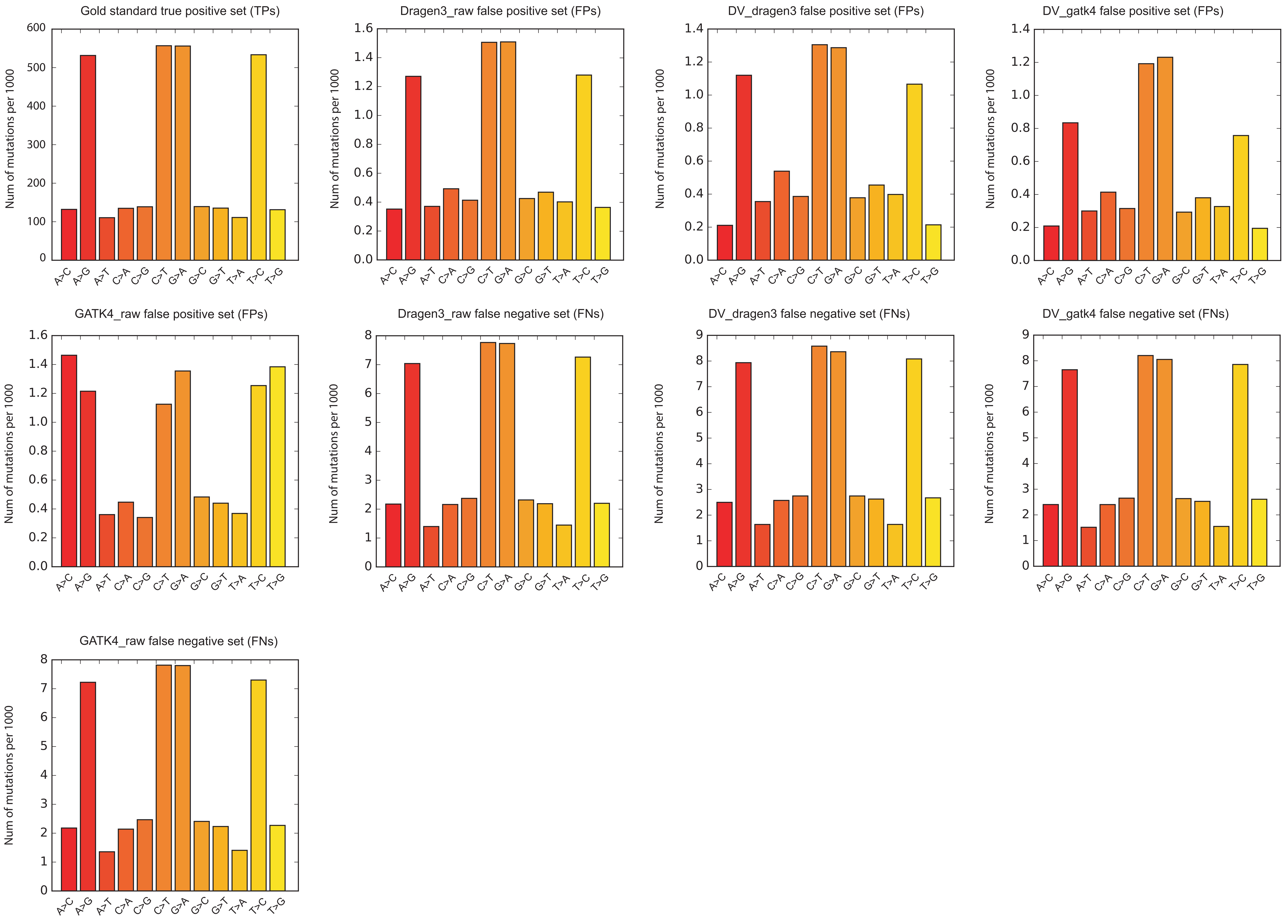

**Figure S6C.** The distribution of substitution signature of false positive and negative variants for the “simulated” dataset (random mutation profile)

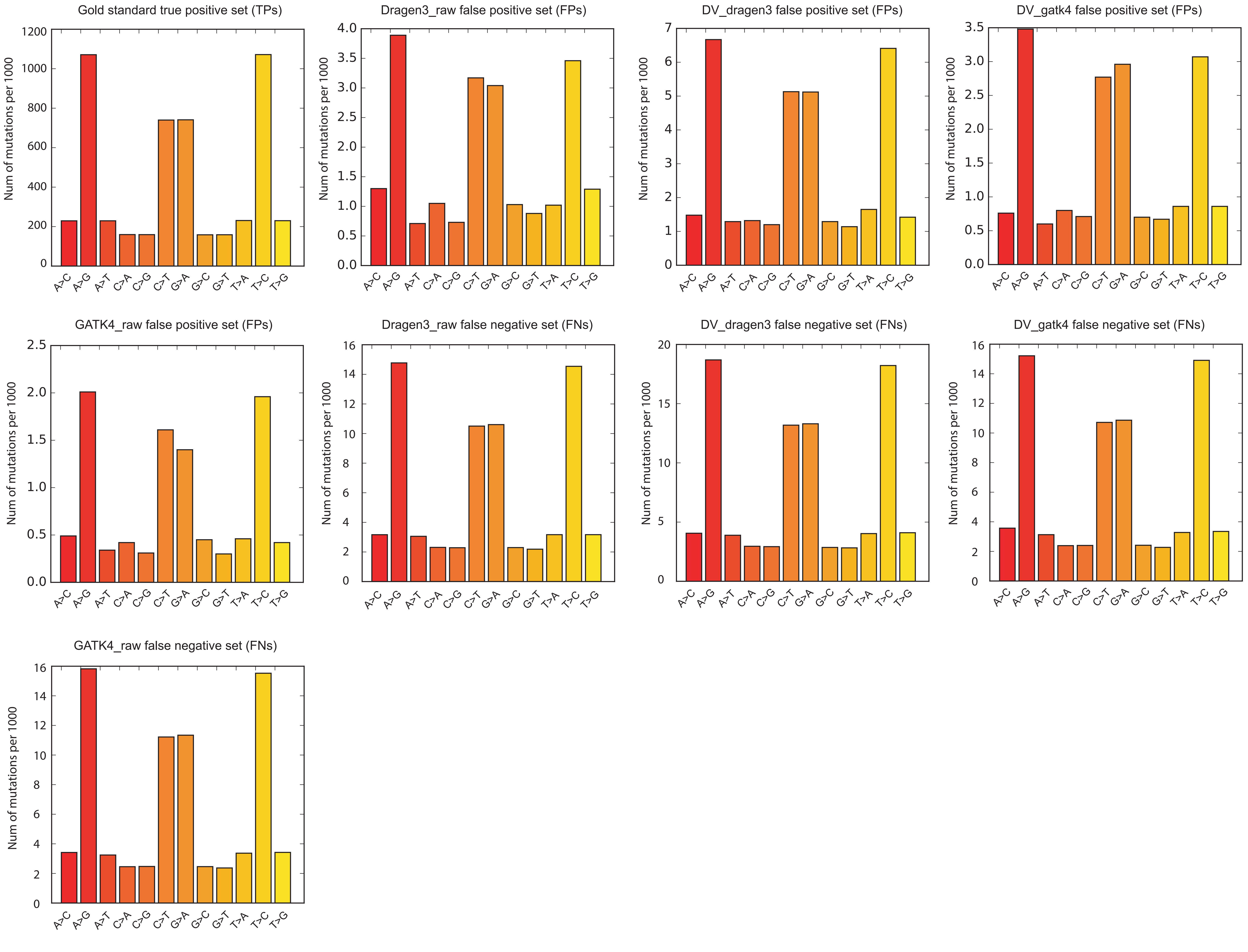

**Figure S6D.** The distribution of substitution signature of false positive and negative variants for the “simulated” dataset (userdefined mutation profile)

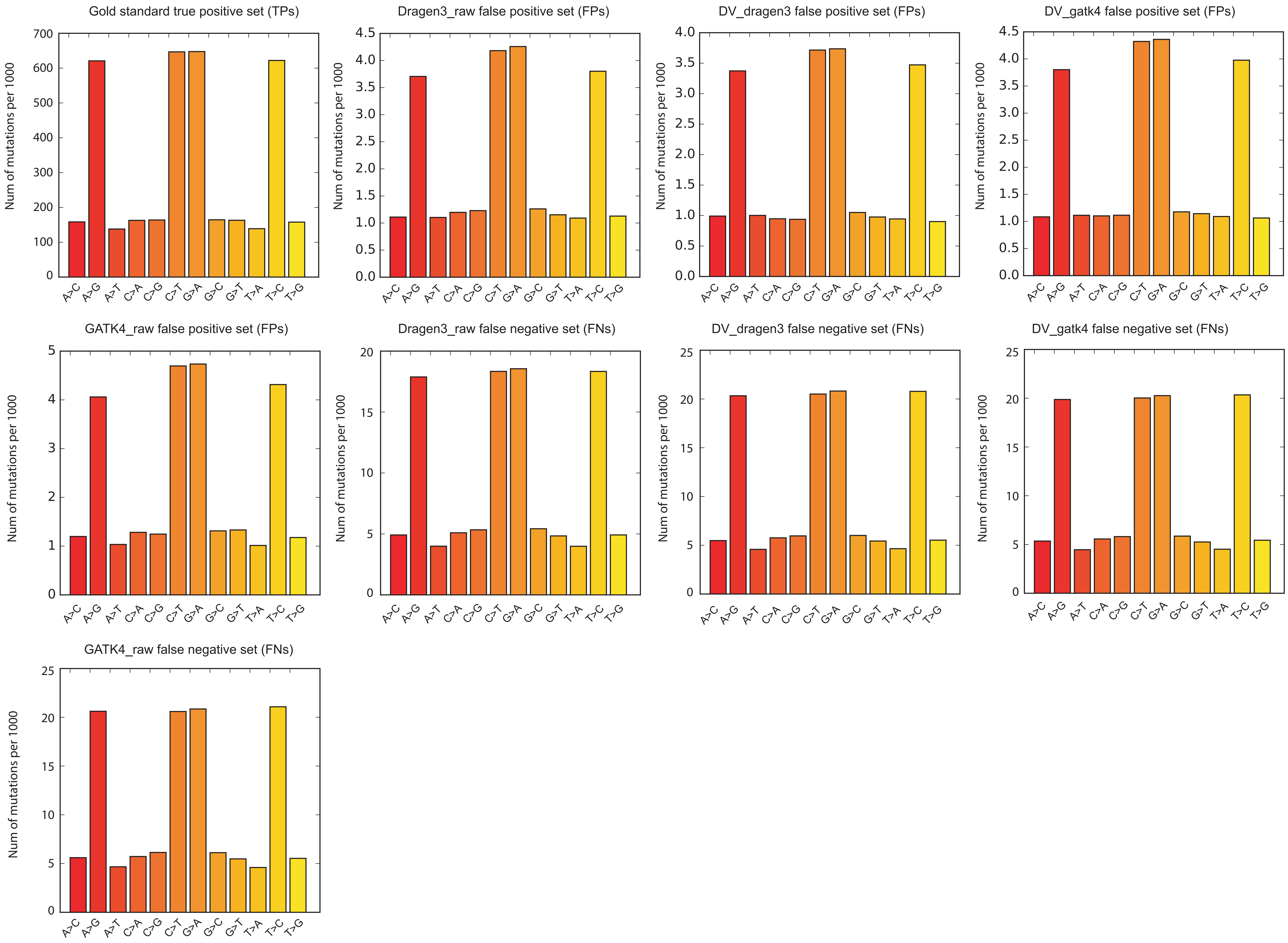

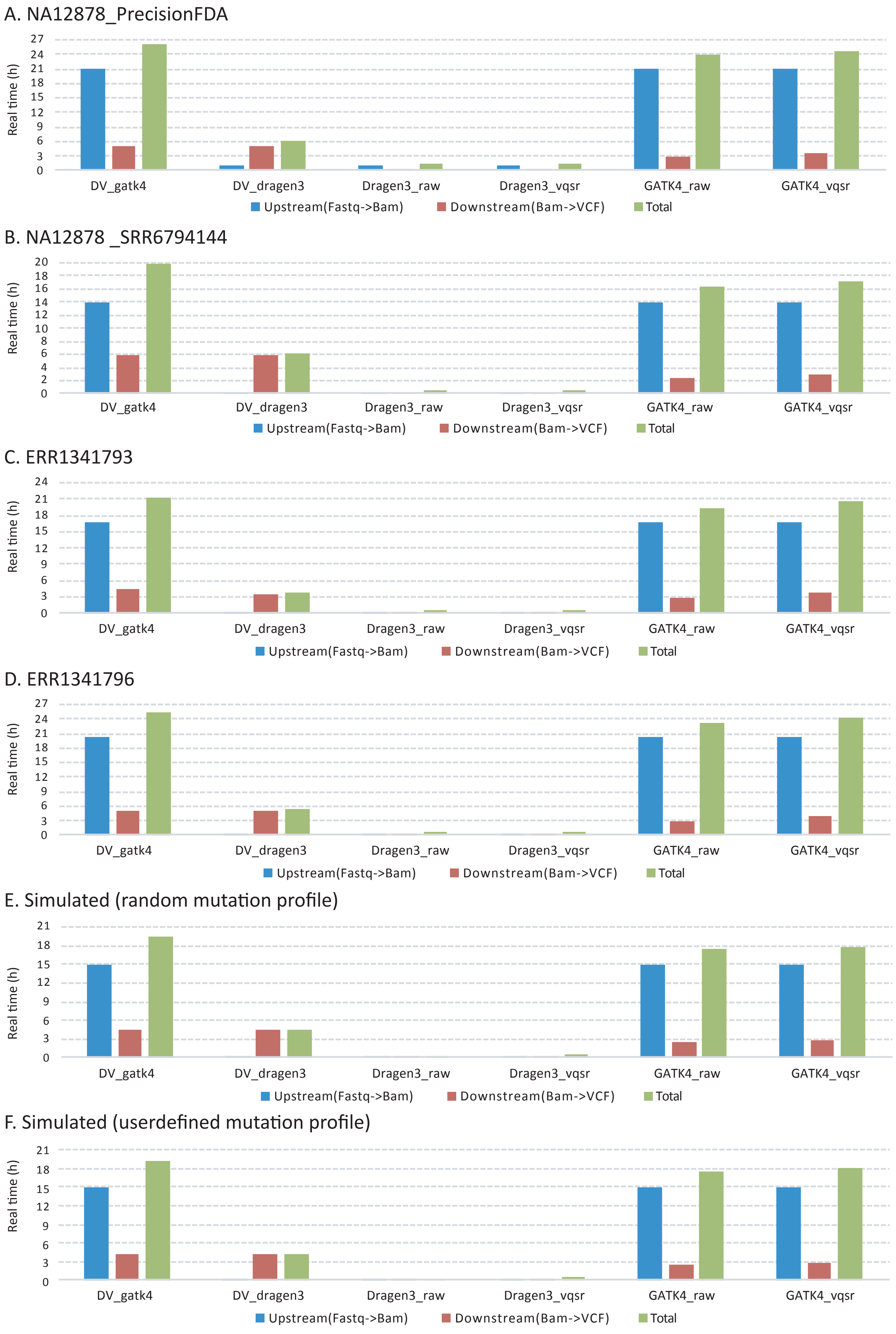


**Figure S7.** Variant calling runtime of six pipeline combinations (*DV_gatk4, DV_dragen3, Dragen3_raw, Dragen3_vqsr, GATK4_raw* and *GATK4_vqsr*) benchmarked on local virtual machine (VM). (A and B – NA12878_PrecisionFDA and NA12878_SRR6794144 datasets; C and D – "synthetic-diploid" ERR1341793 and ERR1341796 datasets; E and F - simulated data based on a random and a userdefined mutation profiles).
